# CK2 modulates adipocyte insulin-signaling and is up-regulated in human obesity

**DOI:** 10.1038/s41598-017-17809-w

**Published:** 2017-12-14

**Authors:** Christian Borgo, Gabriella Milan, Francesca Favaretto, Fabio Stasi, Roberto Fabris, Valentina Salizzato, Luca Cesaro, Anna Belligoli, Marta Sanna, Mirto Foletto, Luca Prevedello, Vincenzo Vindigni, Romeo Bardini, Arianna Donella-Deana, Roberto Vettor

**Affiliations:** 1 Department of Biomedical Sciences, University of Padua, 35131 Padua, Italy; 2 Department of Medicine, University of Padua, Internal Medicine 3, 35128 Padua, Italy; 3 Center for the Study and the Integrated Treatment of Obesity, Padua Hospital, 35128 Padua, Italy; 4 Department of Neurosciences, University of Padua, 35128 Padua, Italy; 5 Department of Surgical, Oncological and Gastroenterological Sciences, University of Padua, Division of General Surgery, 35128 Padua, Italy

## Abstract

Insulin plays a major role in glucose metabolism and insulin-signaling defects are present in obesity and diabetes. CK2 is a pleiotropic protein kinase implicated in fundamental cellular pathways and abnormally elevated in tumors. Here we report that in human and murine adipocytes CK2-inhibition decreases the insulin-induced glucose-uptake by counteracting Akt-signaling and GLUT4-translocation to the plasma membrane. In mice CK2 acts on insulin-signaling in adipose tissue, liver and skeletal muscle and its acute inhibition impairs glucose tolerance. Notably, CK2 protein-level and activity are greatly up-regulated in white adipose tissue from ob/ob and db/db mice as well as from obese patients, regardless the severity of their insulin-resistance and the presence of pre-diabetes or overt type 2 diabetes. Weight loss obtained by both bariatric surgery or hypocaloric diet reverts CK2 hyper-activation to normal level. Our data suggest a central role of CK2 in insulin-sensitivity, glucose homeostasis and adipose tissue remodeling. CK2 up-regulation is identified as a hallmark of adipose tissue pathological expansion, suggesting a new potential therapeutic target for human obesity.

## Introduction

Insulin plays a major role in glucose metabolism increasing its utilization mostly by adipose tissue (AT) and skeletal muscle, and inhibiting hepatic gluconeogenesis. It promotes tissue glucose uptake through the regulated trafficking of endosomal sorting vesicles containing glucose transporter 4 (GLUT4) (GSVs)^1,2^. Insulin also acts as an essential growth factor for AT formation via recruitment and adipogenic differentiation of specific precursors (preadipocytes). AT is a dynamic and plastic organ able to respond to nutrient excess through adipocyte hypertrophy and hyperplasia^3^. In healthy AT increase, insulin sensitivity is preserved allowing adipocytes to buffer the fuel overload^4^. Conversely, AT pathological expansion is characterized by reduced angiogenesis, hypoxia, fibrosis and infiltration of several immune cells, in particular M1 macrophages^5,6^, causing a systemic low-grade chronic inflammation and insulin resistance (IR) that is strongly associated with a reduced adipocyte insulin sensitivity and changes in the pattern of adipokine secretion^7^. Dysfunctional insulin signaling and anomalous AT expansion result in a lipid spillover inducing peripheral lipotoxicity, which is partially causative of obesity complications. Adiposopathy is therefore tightly associated with metabolic alterations as type 2 diabetes (T2D) as well as carcinogenesis and cancer progression^8^.

Insulin binding to its cellular receptor causes the phosphorylation of the insulin receptor substrate 1, which acts as a platform for the assembly of a signaling node including the lipid kinase phosphatidylinositol 3-kinase (PI3K). PI3K generates the phosphatidylinositol 3,4,5-trisphosphate (PIP_3_), which induces the translocation to the plasma membrane of the Ser/Thr protein kinase Akt (also called PKB)^9^. Membrane Akt, once activated by phosphorylation at Thr308 and Ser473, orchestrates a complex metabolic program, leading to cell glucose uptake, increased glycogen and protein synthesis, and inhibition of gluconeogenesis^10^.

Akt1, Akt2 and Akt3, are homologous isoforms expressed in a tissue-dependent manner; adipocytes express mainly Akt2 and at lesser extent Akt1, both involved in the regulation of insulin-mediated metabolic pathways^11^. Akt2 knockout mice^12^ and patients carrying Akt2 loss-of-function mutations^13^ develop severe IR and T2D. Mutations that activate Akt2 cause a left-side overgrowth and severe hypoglycemia^14^ suggesting a central role for Akt signaling in tissue development and in human insulin sensitivity.

Protein kinase CK2 (formerly known as casein kinase 2**)** is a ubiquitous and constitutively active Ser/Thr kinase, usually present as a tetrameric holoenzyme composed of two catalytic (α and/or α’) and two regulatory (β) subunits^15^, which phosphorylates a huge number of protein substrates implicated in several fundamental cell processes^16^. CK2 protein is abnormally elevated in a wide variety of tumors, where it plays a global role as an anti-apoptotic and pro-survival agent^17,18^ operating as a cancer driver^15^.

CK2 has been described to inhibit insulin-release in pancreatic β-cells^19,20^ and to regulate carbohydrate metabolism^21^ and insulin signaling at different levels. In fact, CK2 phosphorylates Akt1 at Ser129 potentiating its activity by maintaining a high extent of phospho-Thr308^22,23^ and inhibits the phosphatase PTEN (Phosphatase and TENsin homologue)^24^, which acts as the main negative regulator of PI3K/Akt signaling by dephosphorylating PIP_3_^25^. It is noteworthy that PTEN is one of the most common somatically mutated proteins implicated in T2D. In mice lacking one copy of *Pten*, PI3K/Akt activation is associated with increased insulin sensitivity^26^ and patients with PTEN loss-of-function mutations show a constitutive insulin sensitivity and obesity^27^.

Here we study the CK2 role in the insulin-signaling of AT, skeletal muscle and liver, demonstrating its positive effect on adipocyte glucose-uptake in cells and on glucose-tolerance *in vivo*. Our data highlight an anomalous CK2 up-regulation in white adipose tissues (WAT) of obese mice and patients, which is independent of the insulin resistance severity or the presence of T2D and is normalized by weight loss.

## Results

### CK2 sustains insulin-stimulated glucose uptake in 3T3-L1 adipocytes

**Figure 1:**
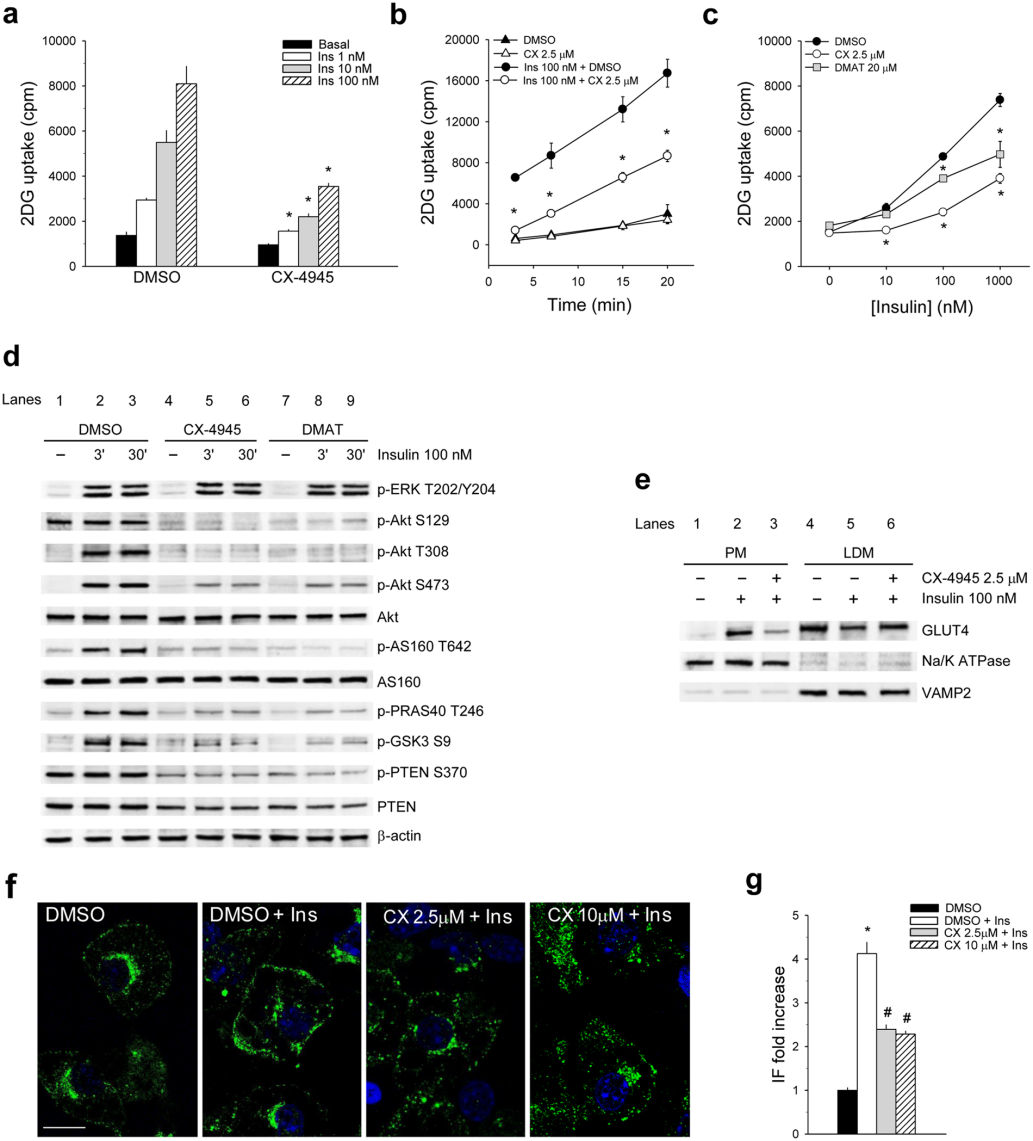
CK2 inhibition reduces insulin-dependent glucose uptake by counteracting Akt signaling and GLUT4 translocation in 3T3-L1 adipocytes. (**a**) 2 Deoxy-D-glucose (2DG)-uptake of 3T3-L1 adipocytes (replicates n = 4) pre-treated for 1 h with DMSO or 2.5 μM CX-4945 and stimulated or not (Basal) with the indicated insulin concentration for 30 min. In 2DG uptake assays total protein content of each sample was quantified and no statistical differences were shown between samples. (**b**) 3T3-L1 adipocytes (n = 4) were pre-treated with DMSO or CX-4945 (CX) for 1 h and stimulated or not with insulin for 30 min. 2DG-uptake was quantified at the indicated times. (**c**) 2DG-uptake of 3T3-L1 adipocytes (n = 4), pre-treated with DMSO, CX-4945 or DMAT for 1 h and stimulated with the indicated insulin concentrations for 30 min. (**d**) 3T3-L1 adipocytes, pre-treated with DMSO, 2.5 μM CX-4945 or 20 μM DMAT for 1 h, were stimulated or not (−) with insulin for 3 or 30 min. Cells were lysed and lysate proteins (40 μg) were analyzed by Wb with the indicated antibodies. β-actin is shown as loading control. (**e**) 3T3-L1 adipocytes, pre-treated with DMSO (lanes 1, 2, 4, 5) or 2, 5 μM CX-4945 (lanes 3, 6), were unstimulated (lanes 1, 4) or stimulated with insulin for 30 min (lanes 2, 3, 5, 6). Plasma membrane (PM, 15 μg) and low density microsome (LDM, 10 μg) fractions were isolated and analyzed by 15% SDS/PAGE and Wb with anti-GLUT4, anti-Na/K ATPase (PM marker) and anti-VAMP2 (LDM marker) antibodies. (**f**) Confocal immuno-fluorescence staining with anti-GLUT4 antibody of 3T3-L1 adipocytes pre-treated with DMSO or CX-4945 for 1 h and then stimulated or not with 100 nM insulin (Ins) for 30 min. Scale bar = 10 μm. Green = anti-GLUT4, Blue = Draq5 for nuclei staining. 100x magnification, zoom factor 2. (**g**) Intensity of fluorescence (IF) of GLUT4 localized at the plasma membrane was measured in at least 30 cells in 3 independent experiments (90 frames) and reported as fold increase relative to DMSO-treated adipocytes. Figure panels are representative of at least four independent experiments. *p < 0.05 *vs* DMSO treatment; ^#^p < 0.05 *vs* insulin-stimulated cells (DMSO + Ins). Results are presented as mean ± SEM.

Preliminary experiments, detailed in Supplementary Results online, demonstrated that CK2 amount and activity were unaffected by insulin stimulation in 3T3-L1 adipocytes (Supplementary Fig. S1). Moreover, we set up the experimental conditions, in which CK2 activity was strongly inhibited in murine and human adipocytes without causing cytotoxic effects (Supplementary Figs S1 and S2). Under these conditions we found that adipose-specific gene expression of mature adipocytes (Supplementary Fig. S3) and adipogenic differentiation (Supplementary Figs S4 and S5) were not significantly affected by CK2-inhibition. Conversely, a great reduction of insulin-induced glucose uptake was observed in 3T3-L1 mature adipocytes, pre-treated for 1 h with the CK2-inhibitor CX-4945^28^ and then stimulated with increasing insulin doses for 30 min (Fig. 1a and Supplementary Fig. S1c). The inhibitor did not influence the basal glucose uptake and the time-course of the insulin-stimulated glucose entry (Fig. 1b). A lower glucose-uptake decrease was obtained using DMAT^29^, a less potent CK2 inhibitor structurally unrelated to CX-4945 (Fig. 1c).

To highlight the molecular mechanisms underlying the glucose-uptake reduction, the effect of CK2 inhibition on the insulin signaling pathways was then examined in 3T3-L1 adipocytes by western blot (Wb) analysis with specific phospho-antibodies (Fig. 1d). CX-4945 and DMAT did not affect the insulin-induced ERK activation as demonstrated by its phosphorylation at Thr202/Tyr204^30^. On the contrary, Akt signaling was greatly counteracted by CK2 inhibitors as indicated by the strong decrease of the constitutive CK2-mediated phosphorylation of Akt1 Ser129 as well as the insulin-induced phosphorylation of Akt1/2 Thr308 and Ser473 (Fig. 1d, lanes 1–3 vs 4–9). Immunostaining with anti-Akt antibodies recognizing Akt1/2/3 isoforms was not modified by cell treatment with inhibitors.

Insulin-activation of Akt1/2 triggered the phosphorylation of its substrates, including AS160 (Akt Substrate of 160 kDa) at Thr642, PRAS40 (Proline-Rich Akt Substrate of 40 kDa) at Thr246 and GSK3β (Glycogen Synthase Kinase 3) at Ser9 (Fig. 1d, lanes 1–3). Consistent with the down-regulation of Akt1/2 activatory sites, CK2-inhibition strongly impaired the phosphorylation of Akt substrates (Fig. 1d, lanes 4–9), including AS160, the most distal insulin-signaling protein linked to GLUT4 translocation^31^, suggesting that CK2 plays a role in the cellular trafficking of this glucose transporter.

CK2 has been described to phosphorylate PTEN^24^ at Ser370 inhibiting its phosphatase activity and degradation^32^. Accordingly, cell treatment with CX-4945 and DMAT caused a substantial decrease of PTEN CK2-phosphorylation at Ser370 and a parallel reduction of its protein level (Fig. 1d, lanes1–3 vs 4–9), suggesting an activation of the phosphatase.

GLUT4 subcellular localization was then examined in CX-4945-pretreated and insulin-stimulated 3T3-L1 adipocytes by isolating the cell plasma membrane (PM), and the low-density microsomes (LDM) (Fig. 1e). In controls, insulin-stimulation increased GLUT4 level in PM fraction (lane 2 vs 1), lowering its amount in LDM fraction, where GLUT4 was mainly localized in unstimulated cells (lanes 4, 5). The finding that the pre-treatment with CX-4945 strongly reduced GLUT4 recruitment to PM fraction (lane 3 vs 2) reveals that CK2 mediates the transporter translocation to the plasma membrane. These observations were further confirmed by confocal immunofluorescence microscopy and quantification of GLUT4 signal at the plasma membrane (Fig. 1f,g). The punctuated perinuclear GLUT4-staining visible under basal conditions moved to PM following insulin-stimulation. Adipocyte pre-treatment with CX-4945 decreased the PM signal enhancing GLUT4 intracellular localization.

These results highlight a novel CK2 function in supporting insulin-signaling activation, GLUT4 recruitment to the cell surface and glucose-uptake in 3T3-L1 mature adipocytes.

### CK2 inhibition reduces insulin-induced glucose uptake and Akt signaling in human adipocyte primary cultures

**Figure 2:**
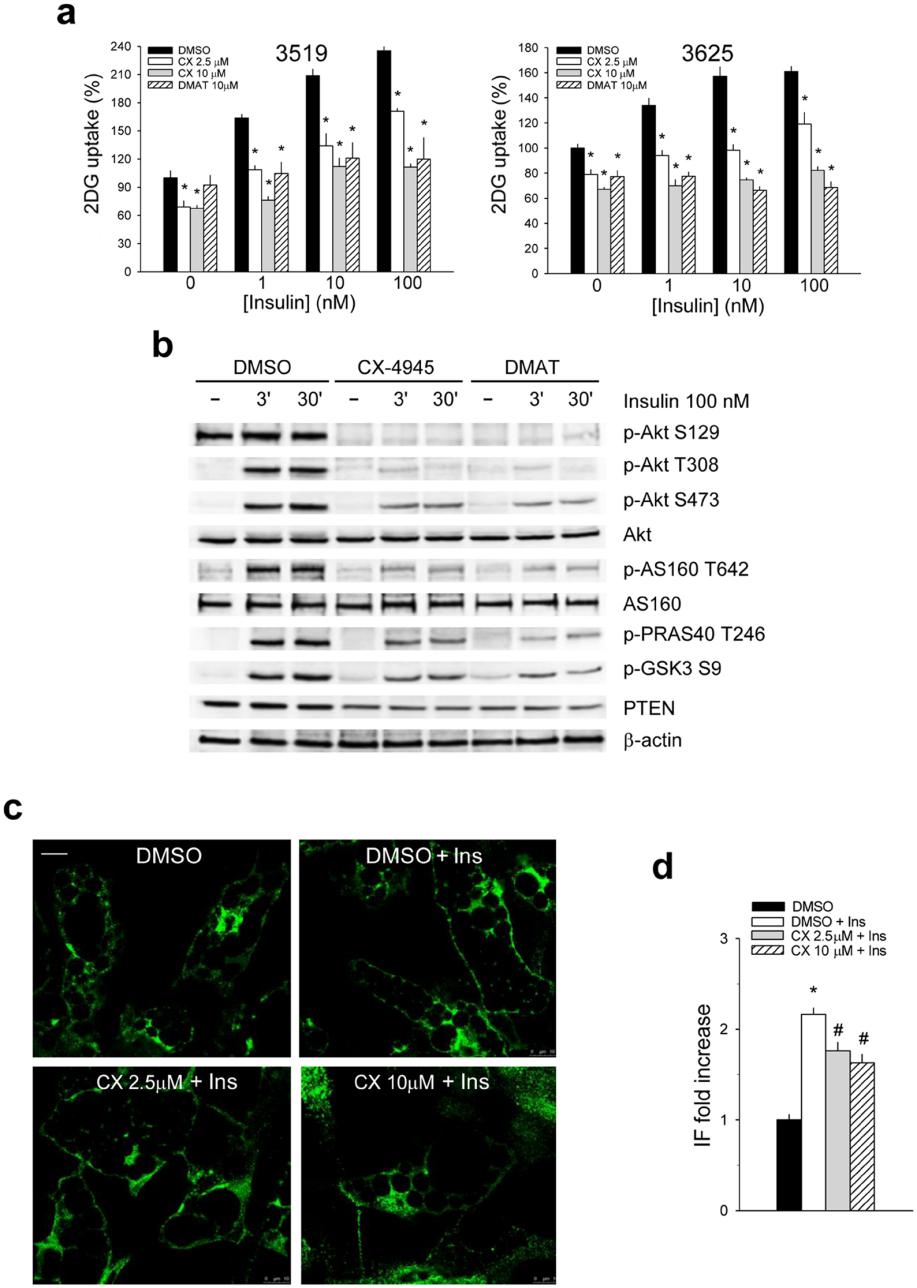
CK2 inhibition reduces insulin-dependent glucose-uptake by counteracting Akt signaling and GLUT4 translocation in human adipocyte primary cultures. (**a**) *In vitro* differentiated mature adipocytes from subcutaneous adipose tissue of patients 3519 and 3625 were pre-treated with DMSO, CX-4945 or DMAT for 1 h, stimulated with different insulin concentrations for 30 min and assayed in 2DG-uptake (n = 4). Data are normalized for total protein content and reported as percentage relative to control adipocytes (DMSO). Similar results were obtained with other 2 patients. (**b**) Human adipocytes, pre-treated for 1 h with DMSO, 2.5 μM CX-4945 or 20 μM DMAT were stimulated or not (−) with insulin as indicated. Lysate proteins (40 μg) were analyzed by Wb. (**c**) Confocal immunofluorescence staining with anti-GLUT4 antibody of human adipocytes pre-treated with DMSO or CX-4945 for 1 h and stimulated or not with 100 nM insulin (Ins) for 30 min. Scale bar = 10μm. Green = anti-GLUT4. 100x magnification, zoom factor 1. (**d**) Intensity of fluorescence (IF) of GLUT4 localized at the plasma membrane was measured in at least 30 cells in 3 independent experiments (90 frames) and reported as fold increase relative to DMSO-treated adipocytes. Panels b, c and d are representative of experiments performed with adipocytes obtained from three different patients. *p < 0.05 *vs* DMSO treatment; ^#^p < 0.05 *vs* insulin-stimulated cells (DMSO + Ins). Results are presented as mean ± SEM.

Results obtained in 3T3-L1 cells were confirmed in human adipocyte primary cultures, where CX-4945 and DMAT pre-treatment substantially inhibited the insulin-stimulated glucose uptake (Fig. 2a). Moreover, also in human adipocytes CK2-inhibition correlated with a striking decrease of the insulin-induced phosphorylation of the Akt1/2 activatory sites (Ser129, Thr308 and Ser473) and substrates (AS160, PRAS40 and GSK3β). PTEN protein-level was substantially reduced, suggesting its activation (Fig. 2b). Finally, immunofluorescence analysis (Fig. 2c) and quantification (Fig. 2d) confirmed the inhibitory effect of CX-4945 on the insulin-induced GLUT4 translocation to the plasma membrane in human adipocytes.

### CK2 activity is required to maintain normal glucose tolerance

**Figure 3:**
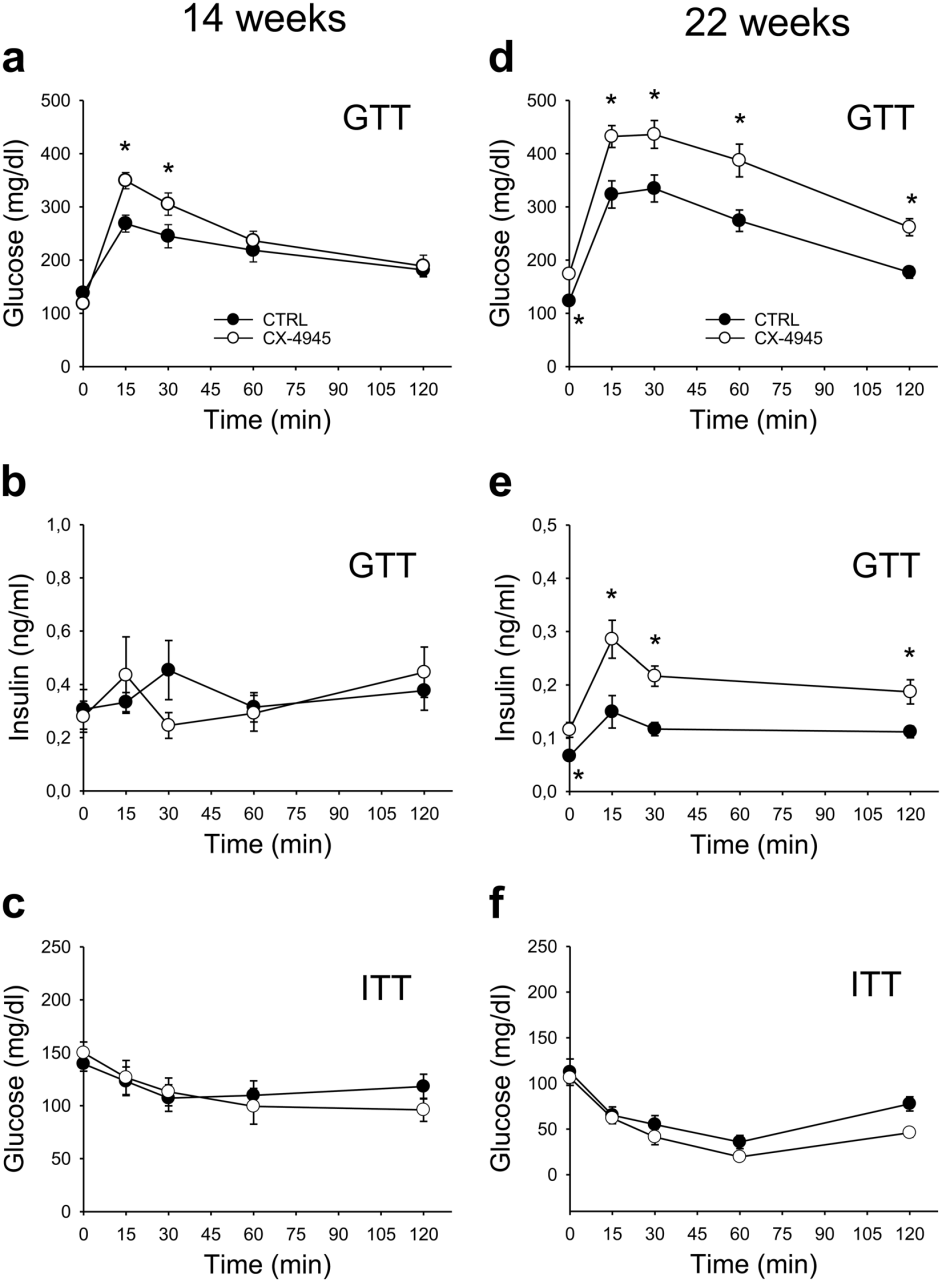
*In vivo* CK2 inhibition induces glucose intolerance. (**a**–**f**) intraperitoneal (ip) glucose tolerance test (GTT, 1.5 g/kg of glucose) (**a**,**b**,**d**,**e**) and insulin tolerance test (ITT, 1U/kg of insulin) (**c**,**f**) were performed in C57BL6/6 J (B6) male mice untreated (CTRL) or 2h-pre-treated with 20 mg/kg CX-4945 in NaCl 0.9% by ip injection at 14 (**a**,**b**,**c**) or 22 weeks of age (**d**,**e**,**f**). Blood glucose (**a**,**c**,**d**,**f**) or insulin (**b**,**e**) levels were then quantified. Experiments were performed with 16 (**a**), 8 (**b**,**f**) or 10 (**c**,**d**,**e**,) mice per group *p < 0.05 *vs* CTRL mice. Results are presented as mean ± SEM.

To investigate the CK2 role in glucose homeostasis and insulin sensitivity *in vivo*, we performed glucose and insulin tolerance tests (GTT and ITT) in 14- and 22-week old B6 wild type mice pre-treated for 2 h with a single intraperitoneal (ip) injection of CX-4945 (20 mg/kg). Blood glucose level was significantly increased during GTT (at 15 and 30 min) in 14-week old mice compared to controls (Fig. 3a), while blood insulin level was unchanged (Fig. 3b). Interestingly, the glucose tolerance decrease induced by CK2-inhibition was more evident and prolonged in 22-week old mice, which showed also a significant higher basal glycemia (Fig. 3d). Moreover, in these animals CX-4945 treatment caused an enhancement of basal and glucose-induced insulin levels compared to controls (Fig. 3e). Conversely, the blood glucose level is not significantly different during ITT in control and CX-4945-treated mice at 14- and 22-weeks of age (Fig. 3c,f), and also in mice treated with higher CX-4945 dose (40 mg/kg, data not shown).

These data show that acute CK2-inhibition induces in mice a transient insulin-resistance in response to glucose load as indicated by a blood insulin-increase unable to normalize glycemia level.

### CK2 acts *in vivo* on insulin signaling in WAT depots, skeletal muscle and liver

CK2 expression and activity were first compared in mouse insulin-target tissues. Quantification of CK2α and CK2β amounts (Supplementary Fig. S6a,b), and of CK2 activity (Supplementary Fig. S6c) showed that white (WQ) and red (RQ) quadriceps and liver contained the highest quantity of this kinase. Lower CK2 protein-level and activity were detected in brown AT (BAT), SAT and epididymal VAT (VAT-E).

**Figure 4:**
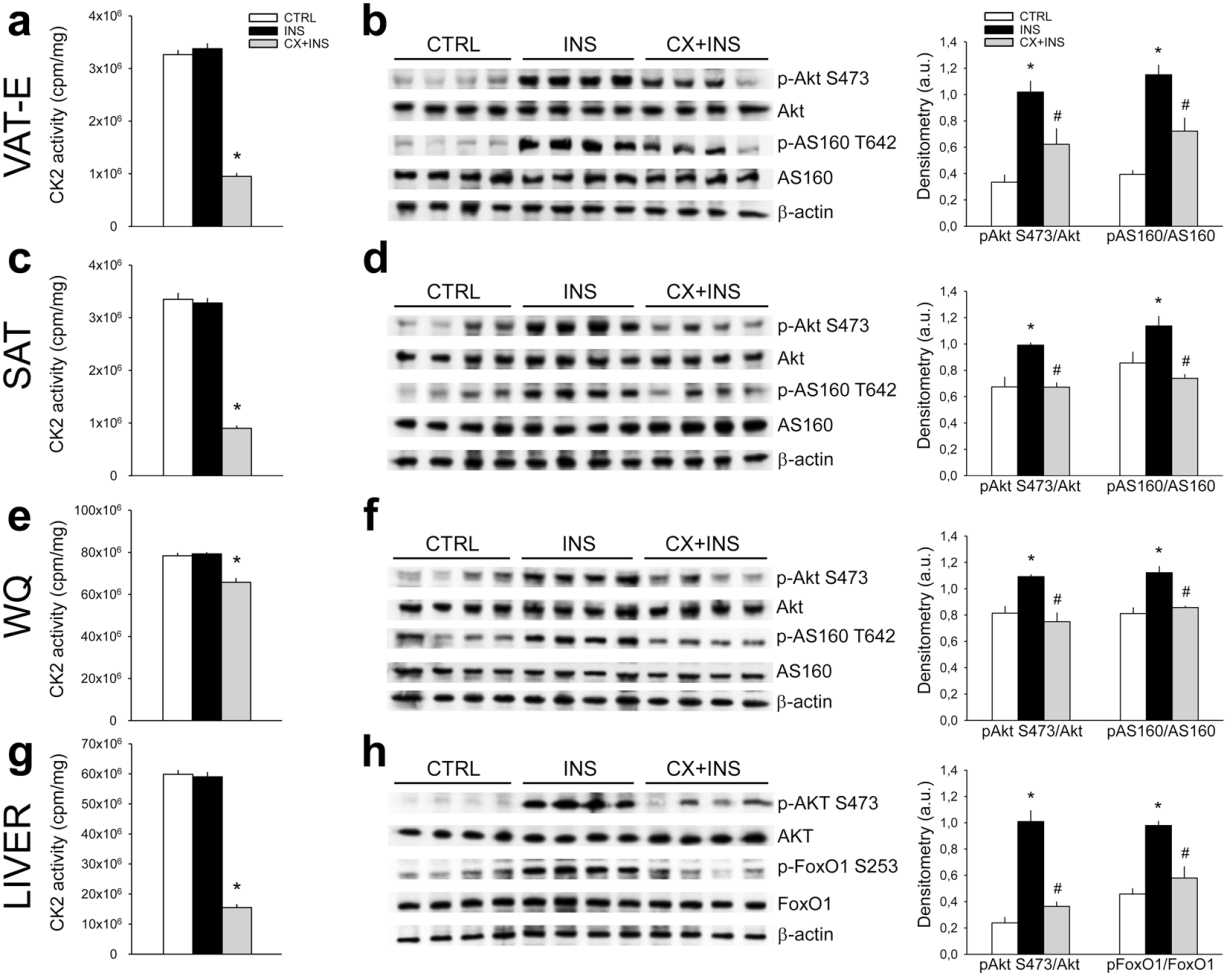
*In vivo* CK2 inhibition counteracts insulin-induced Akt-signaling in mouse adipose tissues, skeletal muscle and liver. (**a**–**h**) C57BL6/6 J (B6) male mice of 22 weeks of age were untreated (CTRL, n = 4), ip injected with insulin (1U/kg) (INS, n = 4) or ip injected with CX-4945 (20 mg/kg in NaCl 0.9%) and the insulin-stimulated after 2 h (CX + INS, n = 4). Thirty min upon insulin-injection mice were sacrificed and the following tissues were collected and lysed: visceral epididimal (VAT-E) (**a**,**b**) and subcutaneous (SAT) (**c**,**d**) adipose tissues, white quadriceps (WQ) (**e**,**f**) and liver (**g**,**h**). (**a**,**c**,**e**,**g**) CK2 activity was assayed in tissue extracts and expressed as cpm of ^33^Pi transferred to the substrate/mg proteins. (**b**,**d**,**f**,**h**) Extract proteins were analyzed by Wb using 40 μg (VAT-E, SAT), 8 μg (WQ) and 10 μg (liver) (left panels). Immunostained bands were quantified by densitometry and right panels reported the mean values ± SEM relative to the phosphorylation extent of Akt S473, AS160 T642 and FoxO1 S253. *p < 0.05 *vs* controls (CTRL); ^#^p < 0.05 *vs* insulin-injected mice (INS). Results are presented as mean ± SEM.

To analyze the effect of *in vivo* CK2 inhibition, mice were acutely pre-treated for 2 h with a single dose of CX-4945, ip-injected with insulin and sacrificed for tissue collection after 30 min following insulin stimulation. CK2 activity and Akt-signaling were then evaluated in insulin-target tissues. Insulin-treatment did not affect the basal kinase activity of different tissues, while CX-4945 injection strikingly inhibited CK2 in VAT-E, SAT and liver (Fig. 4a,c,g). A significant lower activity inhibition was detected in WQ (Fig. 4e) and RQ (data not shown) skeletal muscles. Accordingly, CX-4945-treatment counteracted insulin-trigged Akt1/2 activation in all tissues analyzed as demonstrated by Wb and quantification of Akt Ser473 phosphorylation extent (Fig. 4b,d,f,h). Moreover, CK2 inhibition caused the expected drop of AS160 phosphorylation in VAT-E, SAT and WQ (Fig. 4b,d,f), suggesting reduced GLUT4 translocation, while it was associated with a substantial decrease of FoxO1 Ser253 phosphorylation in liver (Fig. 4h), indicating gluconeogenesis activation. In fact, insulin-triggered Akt-activation induces the inhibitory phosphorylation of FoxO1 Ser253 turning off the transcription of hepatic gluconeogenesis genes^33^. Interestingly, insulin-activation and CX-4945-inhibition of Akt1/2 signaling were more evident in VAT-E and liver (Fig. 4b,h).

These data show that an acute CX-4945 treatment of mice causes inhibition of CK2 activity in insulin-target tissues, ensuing a relevant inactivation of the tissue-specific insulin-driven pathways.

### CK2 is up-regulated in VAT-E and SAT of ob/ob and db/db mice

**Figure 5:**
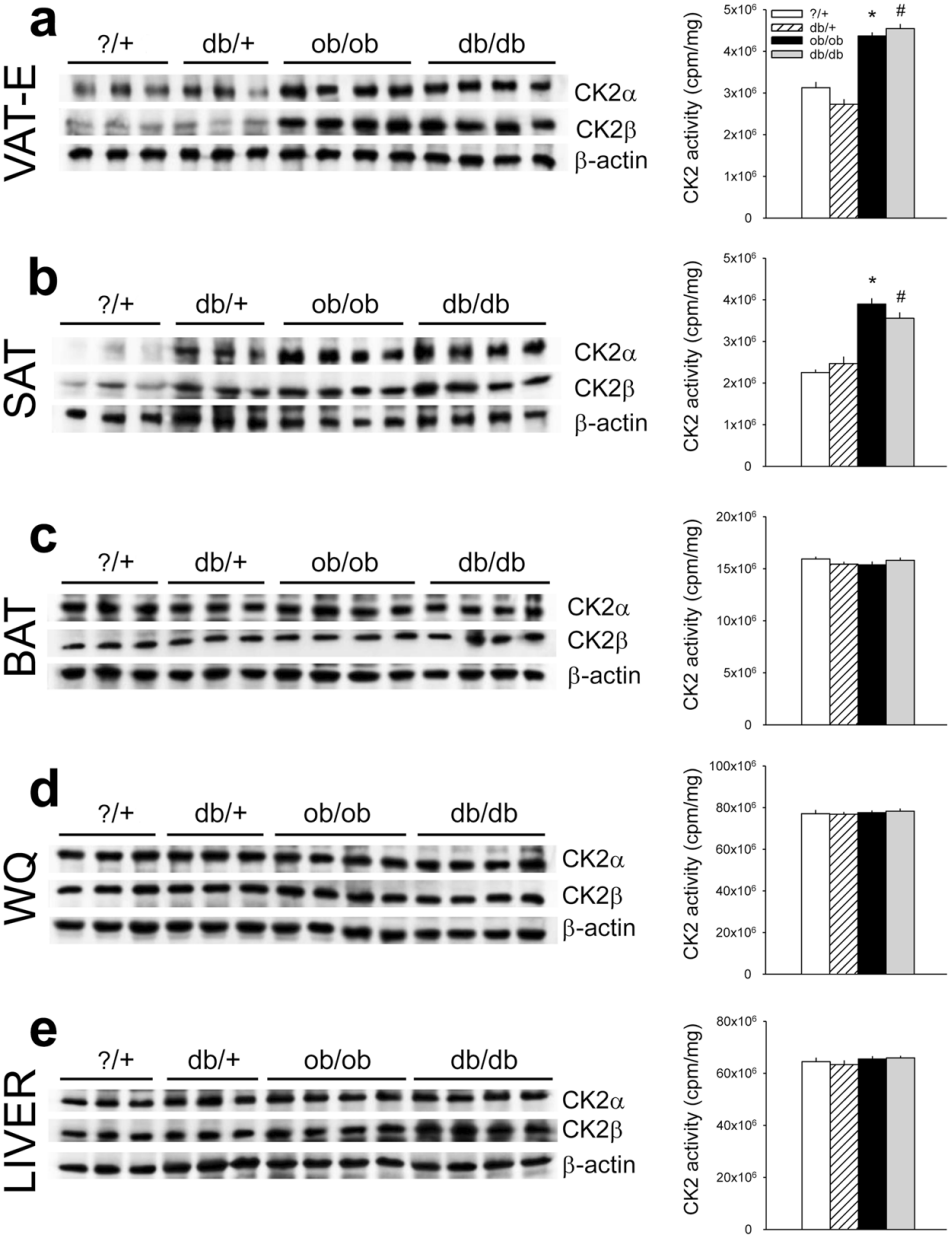
CK2 is up-regulated in white adipose tissue (VAT-E and SAT) of ob/ob and db/db mice. (**a–e**) B6.V-LEP OB/J (ob/ob) and B6.V-LEP OB/J +/? (?/+) control male mice (13 weeks) and BKS.CG-M+/+ LEPRDB/J (db/db) and BKS.CG-M DB/+ (db/+) control male mice (14 weeks) were starved for 6 h and sacrificed to collect and homogenize VAT-E (**a**), SAT (**b**) and BAT (**c**), white quadriceps (WQ) (**d**) and liver (**e**). CK2α and CK2β protein amount (left panels) were analyzed by Wb using 40 μg (**a**,**b**), 20 μg (**c**), 8 μg (**d**) and 10 μg (**e**) of tissue extracts. CK2 kinase activity was also detected (right panels) (n = 5 per group). *p < 0.05 *vs *?/+ ; ^#^p < 0.05 *vs* db/+ . Results are presented as mean ± SEM.

CK2α and CK2β amounts, and CK2 activity were then examined in AT depots, WQ and liver of ob/ob and db/db mice, characterized by severe obesity with different degree of hyperglycemia, hyperinsulinemia and β-cell function^34^ (Fig. 5). In the tissues of the ?/+ and db/+ control mice, CK2 activity was similar to that found in wild type mice (Figs 4, 5 and Supplementary Fig. S6). Notably, higher CK2 protein-level and activity were observed in WAT depots (VAT-E and SAT) of ob/ob and db/db mice compared to the relative controls (Fig. 5a,b), while the protein kinase was unchanged in BAT, skeletal muscle and liver. The finding that CK2 up-regulation was similar in ob/ob and db/db mice suggests that CK2 alterations are associated with WAT expansion in obesity.

### CK2 in obese and diabetic patients: role of weight loss

**Figure 6:**
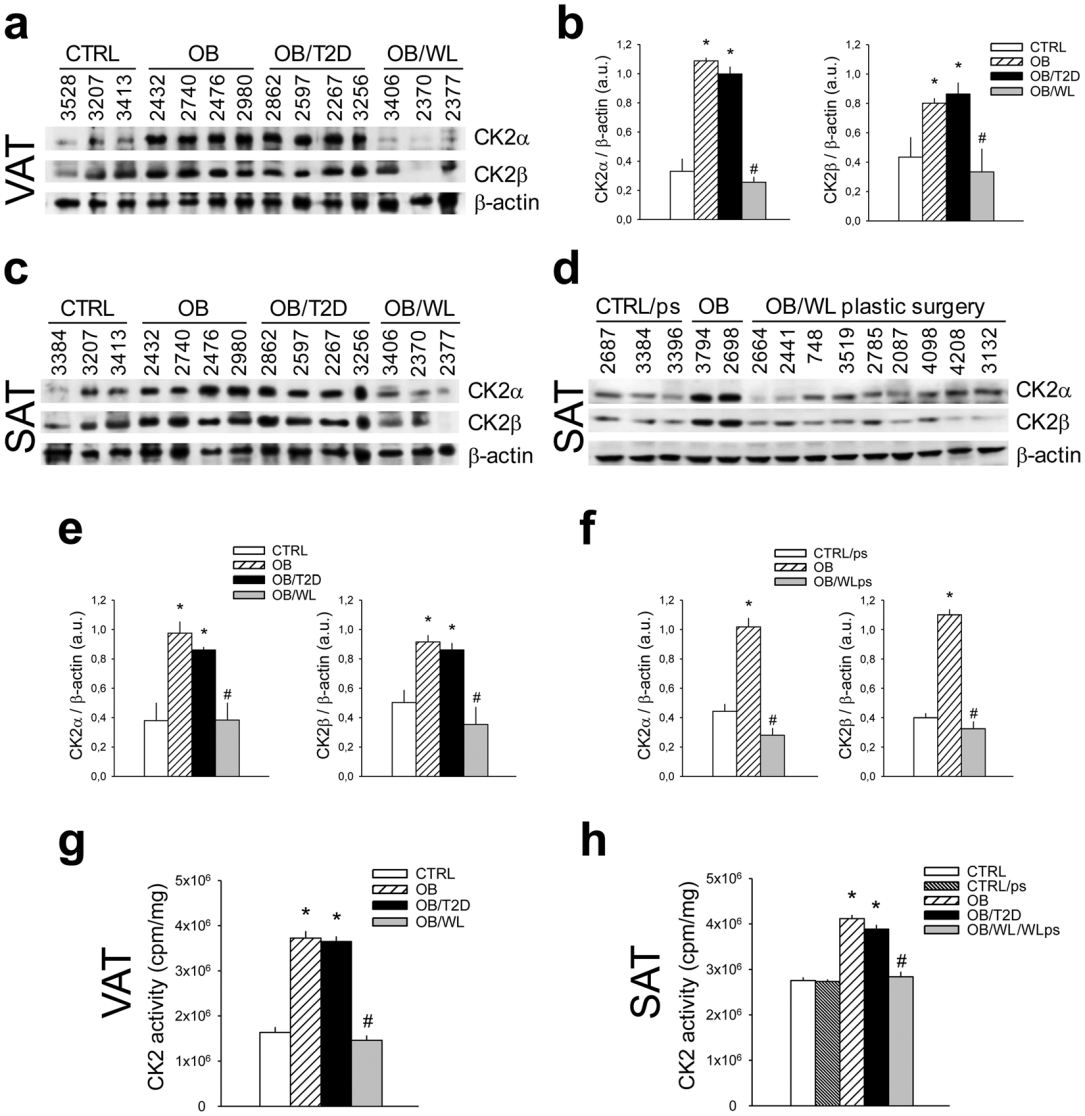
CK2 is up-regulated in abdominal VAT and SAT of human obese and obese/diabetic patients and decreases to normal level upon relevant weight loss. (**a**–**h**) VAT (**a**,**b**,**g**) and SAT (**c–f**,**h**) specimens were obtained from normal-weight subjects, who underwent abdominal surgery (CTRL) or plastic surgery (CTRL/ps), obese (OB), obese/diabetic (OB/T2D) and obese patients after relevant weight loss, who underwent abdominal surgery (OB/WL) or plastic surgery (OB/WLps). Patients are indicated by ID number. Tissue extracts (40 μg) were analyzed by Wb (**a**,**c**,**d**) and CK2α and CK2β amount was quantified by densitometry (**b**,**e**,**f**). CK2 activity was assayed in VAT extracts (**g**) from 4 CTRL, 4 OB, 4 OB/T2D, 4 OB/WL and in SAT extracts (**h**) from 4 CTRL, 3 CTRL/ps, 6 OB, 4 OB/T2D, 15 OB/WL/WLps (5 OB/WL and 10 OB/WLps). *p < 0.05 *vs* CTRL or CTRLps; ^#^p < 0.05 *vs* OB and OB/T2D. Results are presented as mean ± SEM.

CK2 relevance was then evaluated in human obesity and AT remodeling. Obese patients (n = 27), who underwent bariatric surgery and were characterized by different metabolic phenotypes (Supplementary Table S1), were enrolled and their abdominal VAT and SAT were compared with those of 11 normal-weight non-diabetic controls. A great up-regulation of CK2 protein-level and activity was observed in both VAT (Fig. 6a,b,g) and SAT (Fig. 6c–f,h) from obese patients. CK2 hyper-activation was similar in obese subjects (OB) and obese/diabetic patients (OB/T2D). Importantly, we analyzed the VAT and SAT biopsies of 3 obese patients (3406, 2370, 2377), who underwent an abdominal surgery after a substantial weight loss due to bariatric surgery (OB/WL). These patients showed CK2 expression and activity in WAT depots similar to those of normal-weight controls. This observation was confirmed in other 9 SAT biopsies of obese patients, who underwent plastic surgery after relevant weight loss due to bariatric surgery or diet intervention (OB/WLps) (Fig. 6d,f,h) (Supplementary Table S2). CK2 activity was similar in SAT controls obtained from abdominal (CTRL) or plastic (CTRL/ps) surgery, excluding any bias due to different surgery approaches (Fig. 6h).

These data prove that CK2 undergoes an anomalous up-regulation in human obesity, which normalizes after AT reduction and remodeling.

### CK2 in obese and diabetic patients: role of glycemic profile, IR and anti-diabetic therapy

**Figure 7:**
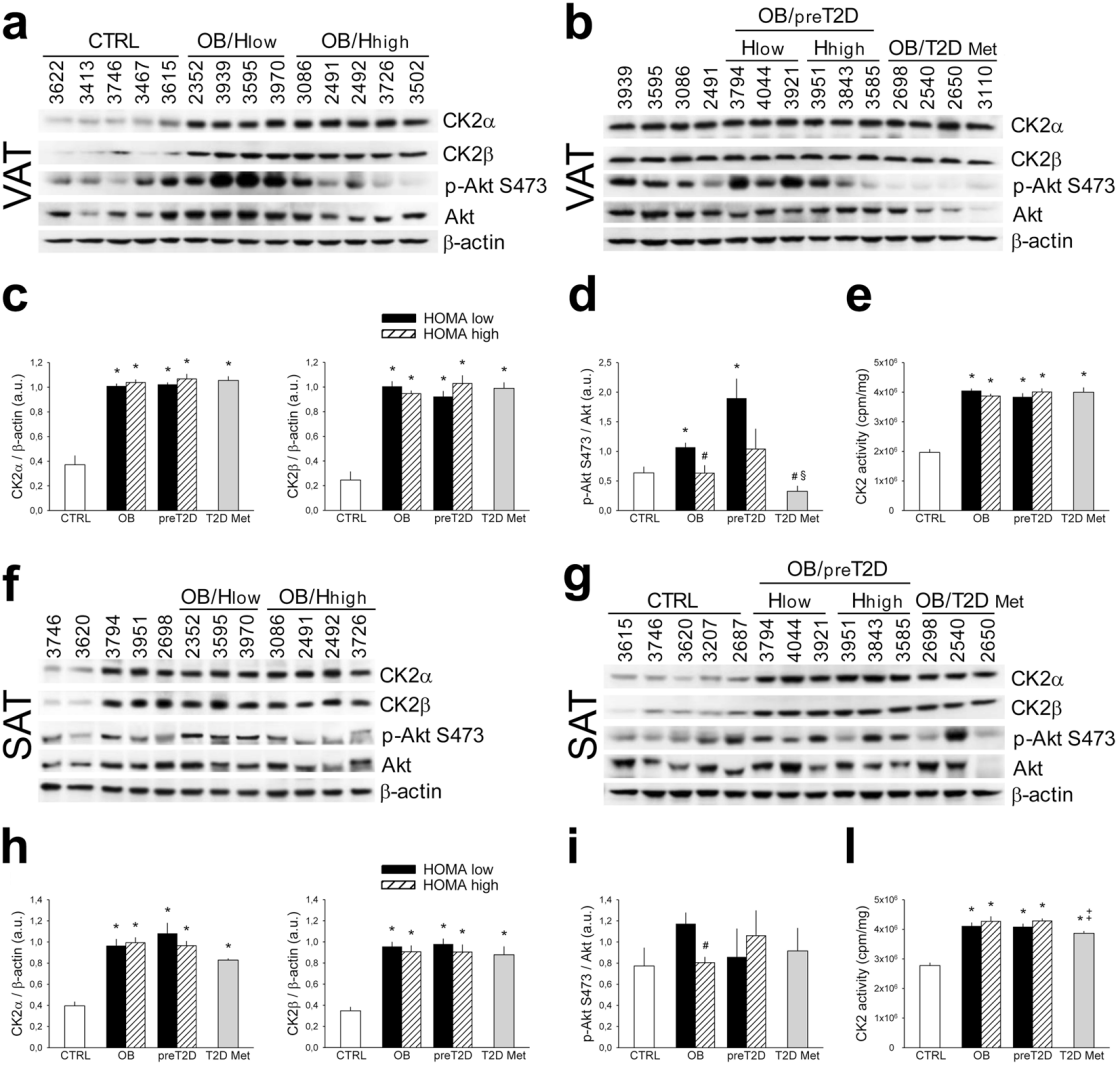
CK2 is up-regulated in abdominal VAT and SAT of human obese patients independently of insulin resistance. (**a**–**l**) VAT (**a**–**e**) and SAT (**f**–**l**) specimens were obtained from normal weight (CTRL), obese (OB) and obese pre-diabetic (OB/preT2D) patients characterized by different HOMA index (Hlow and Hhigh, see Supplementary Table S1) and from obese/diabetic patients treated with oral antidiabetic drug Metformin (OB/T2D Met), indicated by the ID number. To compare several specimens, tissue extracts 3939, 3595, 3086 and 2491 were present in both panels a and b, while extracts 3746, 3620, 3794, 3951 and 2698 were present in panels f and g. Tissue extracts (40 μg) were analyzed by Wb (**a**,**b**,**f**,**g**); CK2α and CK2β amount, and Akt S473 phosphorylation extent were quantified by densitometry (**c**,**d**,**h**,**i**). CK2 activity was assayed in VAT extracts (**e**) of 7 CTRL, 5 OB/Hlow, 5 OB/Hhigh, 3 OB/preT2D Hlow, 4 OB/preT2D Hhigh, 4 OB/T2D Met and in SAT extracts (**l**) of 8 CTRL, 4 OB/Hlow, 5 OB/Hhigh, 3 OB/preT2D Hlow, 4 OB/preT2D Hhigh, 5 OB/T2D Met.*p < 0.05 *vs* CTRL; ^#^p < 0.05 *vs* OB/Hlow; ^§^p < 0.05 *vs* OB/preT2D/Hlow; ^‡^p < 0.05 *vs* OB/Hhigh and OB/preT2D/Hhigh. Results are presented as mean ± SEM.

To analyze the potential role of CK2 in the molecular mechanisms involved in IR genesis and maintenance, we studied obese and obese/diabetic patients with different glycemic profile and degree of IR estimated by HOMA index (Supplementary Table S1). Obese patients, who underwent bariatric surgery, were classified as normoglycemic (OB), prediabetic (OB/preT2D) or diabetic (OB/T2D Met) according to their basal glycemia, OGTT response, medical history and therapy. OB and OB/preT2D groups were divided in 2 subgroups with low or high HOMA, considering 3 as cut off (Hlow or Hhigh). Furthermore, only metformin-treated patients were included in OB/T2D Met group to avoid the potential misleading effect of the insulin-treatment on CK2. We quantified CK2α and CK2β protein-level as well as Akt S473 phosphorylation as a marker of peripheral insulin-sensitivity in VAT (Fig. 7a–d) and SAT (Fig. 7f–i) of patients. By loading the same reference samples in different gels we could compare several human specimens (Fig. 7 and Supplementary Fig. S7a–d). CK2 activity was also evaluated in all samples (Fig. 7e,l). Up-regulation of CK2 protein-level and activity in VAT and SAT was similar in the different groups of obese patients, independently of the HOMA index and the glycemic status. In VAT, the Akt protein-level and phosphorylation were substantially higher in OB/Hlow patients than in controls (CTRL) and OB/Hhigh subjects (Fig. 7a,d). Moreover, OB/T2D Met patients exhibited a very low level of VAT Akt phosphorylation compared to other subgroups (Fig. 7b,d). Interestingly, a negative correlation was observed in obese patients between VAT Akt phosphorylation extent and fasting insulin level (R = −0.446; p = 0.06) or HOMA (R = −0.488; p = 0.04) (Supplementary Fig. S7e,f). Akt S473 phosphorylation was significantly higher in SAT of OB/Hlow patients compared to OB/Hhigh group (Fig. 7f,i).

Our data demonstrate that AT CK2 up-regulation is an obesity hallmark unaffected by IR or T2D.

## Discussion

Several groups have indicated that CK2 is regulated by insulin in human skeletal muscle, rat liver and cell cultures, however conflicting data have been reported on insulin effect toward CK2 protein-level and activity^21,35–39^. We demonstrate that CK2 amount and activity are unaffected by insulin treatment in adipocyte cultures (Figs 1d, 2b and Supplementary Fig. S1) and in mouse insulin-target tissues after *in vivo* insulin injection (Fig. 4). More importantly, we show for the first time that CK2 is required for insulin-induced glucose uptake, regulating the hormone-signaling at different levels in both murine and human mature adipocytes (Figs 1, 2). In fact, CK2-inhibitors strongly counteract Akt1/2 activation directly by reducing the phosphorylation extent of Akt1 Ser129 and indirectly by stimulating PTEN, the main negative regulator of Akt1/2 activation. The down-regulation of Akt1/2 activity caused by CK2-inhibition is associated with strong impairment of the following events: phosphorylation of the Akt-target AS160, GLUT4-translocation to the cell surface and insulin-induced glucose-uptake. Our results together with the finding that CK2 mediates the GLUT4 depletion induced in 3T3-L1 adipocytes by long-term insulin-stimulation^40^ demonstrate that CK2 is involved in GLUT4 regulation at different levels.

*In vivo* analysis in insulin-stimulated mice demonstrates that an acute pre-treatment with the CK2 inhibitor CX-4945 is sufficient to strongly counteract the activation of tissue-specific insulin-signaling. In particular, our results suggest that CK2-inhibition decreases the glucose-uptake in VAT-E, SAT and skeletal muscle, while activates the gluconeogenesis in liver (Fig. 4). Accordingly, CX-4945-treated mice exhibit significantly higher blood glucose levels than controls during GTT, indicating an impaired glucose tolerance, which is particularly evident in 22- as compared to 14-week old mice (Fig. 3a,d). In old animals, CK2 inhibition induces a transient IR state increasing fasting glucose and insulin levels (Fig. 3d,e). This worsening effect of CX-4945 treatment in older animals suggests a synergistic action of CK2 inhibition and aging in decreasing insulin sensitivity. CK2-inhibition has been reported to increase insulin production and secretion in pancreatic β cells either isolated or *in vivo* ^19,20^. Our results suggest that the enhanced insulin-response to glucose observed in CX-4945-treated old mice could be partially induced by the hyperglycemia associated with CK2-inhibition in several tissues.

As also previously reported^20^, ITT performed in acutely CX-4945-treated and control mice is similar (Fig. 3c,f). Mice are injected with a high dose of exogenous insulin during ITT and skeletal muscle contributes to glucose disposal more than WAT, particularly in lean mice^2^. We could therefore hypothesize that CK2 role in WAT and liver insulin-signaling would be more important than in skeletal muscle.

Our CK2 analysis in mouse models of obesity and metabolic alterations showed that both amount and activity of CK2 are substantially higher in WAT (VAT-E and SAT) of ob/ob and db/db mice than of controls, while they are similar in BAT, muscle and liver (Fig. 5). These results are consistent with a recent report showing that CK2 is preferentially activated in WAT of high-fat diet (HFD) mice or in white adipocytes stimulated with β3 agonist^41^. These authors also showed that chronic CK2 inhibition by CX-4945-treatment for 40 days protects mice from diet-induced obesity and moderately improves insulin sensitivity by promoting UCP-1 dependent thermogenesis. It has also been demonstrated that prolonged CK2 blockage by CX-4945 reduces the diet-induced hyperglycemia and glucose intolerance in HFD mice^20^. The finding that the CX-4945-effect detected in HFD mice during GTT is different from that we measured in wild-type animals, suggests that CK2 could be implicated in processes interfering with glucose-handling and energy-expenditure during obesity development.

CK2 relevance in obesity was further supported by data obtained in obese patients. We found that VAT and SAT from obese subjects exhibit a strong CK2 up-regulation independent of the concomitant presence of T2D (Figs 6, 7). We also studied the potential role of CK2 in IR by analyzing the WAT of obese patients, classified according to their HOMA index and glycemic profile, considering the T2D development in obesity. It is widely accepted that the dynamic phase of obesity is indeed characterized by healthy AT expansion in response to increasing insulin level. When adipocytes reach a critical size and macrophages infiltrate AT giving rise to a local inflammation and changes in adipokines production, fat tissue fails to adequately respond to insulin. In parallel, free fatty acid release increases and contributes to peripheral lipotoxicity inducing IR and favoring T2D development^4^. Our results demonstrate that the WAT CK2 amount and activity observed in the different groups of BMI-matched obese patients are higher than in normal-weight controls, independently of IR severity, early glycemia alterations, overt T2D or different levels of WAT insulin-sensitivity (Fig. 7). Constitutive activation of VAT Akt1/2 signaling is indeed present in fasting obese and obese/pre-diabetic patients characterized by a low HOMA index, suggesting that AT preserves its insulin sensitivity despite the initial alterations in glucose homeostasis. On the contrary, Akt1/2 phosphorylation is low in obese patients with high HOMA index and it is almost absent in diabetic patients (Fig. 7).

It is noteworthy that CK2 up-regulation in WAT of obese patients seems to be tightly linked to AT pathophysiological aspects related to obesity rather than to IR severity and T2D development. CK2 up-regulation occurring during AT expansion might represent an attempt to improve the storage capacity of white adipocytes sustaining their insulin sensitivity and promoting glucose-uptake and utilization. Nevertheless, with obesity progression this putative regulatory mechanism is probably overcome by the occurrence of interfering events favoring the development of IR and T2D.

Pathological white adipocytes show remarkable similarities with tumor cells, which exhibit anomalous CK2 hyper-activation^15,17,18^ and metabolic alterations including increased avidity for glucose^42^. Recently, CK2 has been shown to sustain the elevated energy demand of glioma cells by down-regulation of PDK4-AMPK axis, promoting the glucose entrance and the ATP production required by malignant cell proliferation^43^.

The hypothesis that CK2 up-regulation might be considered as an AT intrinsic dysfunction occurring during obesity is also supported by the finding that the CK2 amount and activity revert to normal levels in WAT of obese patients after a relevant weight loss (Fig. 6). CK2 decrease is evident in all patients regardless of the type of weight loss intervention, the presence of metabolic complications and the achievement of a normal BMI, demonstrating that the fat mass reduction and the related AT functional remodeling are associated with a normal CK2 activity and the improvement of patient metabolic parameters. These data suggest that a proper CK2 down-regulation might be considered as an additional tool to counteract human obesity and that the CK2-inhibitor CX-4945, currently used in oncological clinical trials (NCT02128282)^28^, could be regarded as an emerging drug targeting AT. This hypothesis is also supported by the outcome that CX-4945-treatment reduces diet-induced obesity in mice by promoting whole-body energy expenditure^41^ and that CK2 is involved in pathways implicated also in adiposopathy as HIF1-driven angiogesis^28^ and NF-kB-mediated inflammation^15^.

All together our data unveil a new role of CK2 in promoting AT insulin-signaling and influencing glucose homeostasis. Moreover, we highlight human obesity as a new pathological condition inducing abnormally elevated CK2 activity.

## Methods

### Materials

[γ^33^P]ATP was purchased from Perkin-Elmer (Waltham, MA, USA) and 2-Deoxy-D-^3^H-glucose from GE Healthcare (Waukesha, WI, USA). Protease inhibitor cocktail was from Calbiochem (Darmstadt, Germany), while phosphatase inhibitor cocktails were from Sigma-Aldrich (Dorset, U.K.). CX-4945 was purchased from AbMole BioScience (Hong Kong, China) and the other chemicals were from Sigma-Aldrich. The peptide RRRADDSDDDDD and the inhibitor DMAT^29^ were kindly provided by Dr. O. Marin (University of Padova, Italy) and Dr. Z. Kazimierczuk (Warsaw Life Sciences University, Poland), respectively. Culture medium, additives, Bodipy and Micro BCA Assay Kit were from Thermo Fisher Scientific (Waltham, MA, USA). Insulin (Humulin R) was from Eli Lilly (Italy) and Rosiglitazone from Bertin Pharma (Montigny le Bretonneeux, France). Dexamethasone, IBMX, paraformaldehyde and Immobilon-P membranes were from Sigma-Aldrich (Dorset, U.K.).

### Antibodies

CK2α antisera was prepared as described^44^, anti-p-Akt (Ser129) (catalog ab133458), anti-CK2β (catalog ab76025), anti-GLUT4 used for Wb (catalog ab62375) and anti-VAMP2 (catalog ab3347) antibodies were from Abcam (Cambridge, UK), anti-β-actin (catalog A5441) from Sigma-Aldrich. Antibodies raised against PTEN (catalog sc-7974), Akt1/2/3 (catalog sc-8312) Na/K ATPase (catalog sc-28800) and GLUT4 (catalog sc-1606) used for immunolocalization were from Santa Cruz Biotechnology (Santa Cruz, CA, USA), while anti-phospho-PTEN (Ser370) (catalog 07–889) was from Merck Millipore (Darmstadt, Germany). Anti-p-Akt (Thr308) (catalog #13038), anti-p-Akt (Ser473) (catalog #4060), anti-AS160 (catalog #2670), anti-p-AS160 (Thr642) (catalog #4288), anti-p-PRAS40 (Thr246) (catalog #13175), anti-GSK3β (Ser9) (catalog #5558), anti-p-FoxO1 (Ser253) (catalog #9461), anti-FoxO1 (catalog #2880) antibodies were from Cell Signaling Technology (Danvers, MA, USA).

### Cell cultures

#### 3T3-L1 adipogenic differentiation

1 × 10^5^ 3T3-L1 (mouse preadipocyte cell line, mycoplasma-free from ECACC, Sigma-Aldrich, cat no 86052701) cells/well (24 wells plate) or 5 × 10^5^ cells/well (6 wells plate) were seeded in standard medium: Dulbecco’s Modified Eagle Medium (DMEM) high glucose (4.5 g/l), 2 mM glutamine, 150 U/ml streptomycin, 200 U/ml penicillin and 10% of fetal bovine serum (FBS). Two days post confluence, standard medium was supplemented with 2 µM insulin, 1 µM dexamethasone and 0.5 mM 3-Isobutyl-1-methylxanthine (IBMX) (Adipogenic Medium = AdM) for 3 days and without IBMX for other 6/9 days of adipogenic differentiation. Mature adipocytes were maintained in standard medium for 2 days before further assays.

#### Human adipocyte primary cultures

Human abdominal SAT specimens were minced and digested in a collagenase type II solution (1 mg/ml), centrifuged at 300 g, and red blood cells were removed using standard lysis buffer. 0.5 × 10^6^ cells/well (24 wells plate) from the stromal vascular fraction (SVF) and 1.5 × 10^6^ cells/well (6 wells plate) were seeded in human-standard medium (h-SdM): 10% FBS DMEM F12 supplemented with 150 U/ml streptomycin, 200 U/ml penicillin, 2 mM glutamine, 1 mM HEPES. At cell confluence (1/2 days after), medium was replaced with human-adipogenic medium (h-AdM): DMEM F12 (with 150 U/ml streptomycin, 200 U/ml penicillin, 2 mM glutamine, 1 mM HEPES) containing 66 nM insulin, 100 nM dexamethasone, 1 nM 3,3’,5-triiodio-L-thyronine (T3), 10 µg/ml transferrin, 33 µM biotin, 17 µM pantothenate, 0.25 mM IBMX, 10 μM rosiglitazone. IBMX and rosiglitazone were removed after 3 days of culture. At day 9, insulin, dexamethasone and T3 were removed and cells were washed out 2 days before further analysis.

### Animal care and handling

Mice were purchased from Charles River Laboratories Italia (Lecco, Italy) and the standard diet (4% fat, Crispy Pellets Omnivores) was from Versele-Laga (Deinze, Belgium).

C57BL6/J (B6 n = 77), B6.V-LEP OB/J (ob/ob, n = 5), BKS.CG-M+/+ LEPRDB/J (db/db, n = 5) and controls B6.V-LEP OB/J+/? (?/+, n = 5), BKS.CG-M DB/+ (db/+, n = 5) male mice were fed with standard diet (4% fat) in a temperature and humidity controlled setting with a 12 h light/dark cycle. Body weight (g) of ob/ob mice and their controls (?/+) were 44.1 ± 2.5 and 28.3 ± 2.8, respectively, while their blood glucose level (mg/dl) was 157.4 ± 19.2 and 132.6 ± 18.2. Body weight (g) of db/db mice and their controls (db/+) were 49.3 ± 1.4 and 27.5 ± 1.3, respectively, while their blood glucose level (mg/dl) was 305.5 ± 30.4 and 133.0 ± 5.6. Where indicated B6 mice were ip injected with a bolus of CX-4945 (20 mg/kg) and/or with insulin (1U/kg) diluted in saline solution. ob/ob, db/db and relative controls were starved for 6 h and sacrificed by CO_2_ euthanasia to collect and immediately freeze in liquid nitrogen the indicated tissues.

### Human subjects

Adipose tissue samples (VAT and SAT) were collected from 27 (17 F/10 M, age 48, 2 ± 12,2 years) obese patients (BMI > 38 Kg/m^2^) during bariatric surgery, divided in subgroups on the basis of glycemic profile, HOMA index and antidiabetic therapy (Supplementary Table S1); 11 (8 F/3 M, age 47, 8 ± 14,2 years) nondiabetic, normal-weight subjects (18.5 < BMI < 24.9 kg/m^2^) were enrolled as control group and VAT and SAT were harvested during abdominal surgery as laparoscopic cholecystectomy (n = 5) and fundoplication surgery (n = 3) or SAT alone during plastic surgery (ps) for minor abdominal wall defects (n = 3). From 12 obese patients who had achieved relevant weight loss (≥50% of excess WL) by bariatric surgery (n = 9) or caloric restriction (n = 3) both VAT and SAT were obtained (n = 3) during laparoscopic cholecystectomy performed 1 year after bariatric surgery, only SAT (n = 9) during abdominoplasty for abdominal wall laxity (Supplementary Table S2). SAT of WL/ps patients (ID 3519, 3625, 5806, 5860) were used to isolate the SVF for human adipocyte primary cultures. After sampling, tissues were immediately frozen in liquid nitrogen and stored at −80 °C before further assays.

### Anthropometric and biochemical measurements in human subjects

Weight, eight as well as waist circumference (at midpoint between the lower margin of rib cage and the top of iliac crest) were measured in overnight fasted obese patients. Blood samples were used for the biochemical determinations, performed with standard diagnostic kit: for glucose (Glucose HK Gen.3 Cobas C System, Roche Diagnostic, USA), Insulin (IMMULITE 2000), IL6, TNFα (IMMULITE 1000 Immunoassay System, Siemens Healthcare GmbH, Germany), hsCRP (Cardiophase Flex reagent Cartridge, Dimension Vista, Siemens) and Leptin (RIA – CT, Mediagnost, Germany). HOMA was calculated and used as IR index^45^.

### Glucose uptake assay

After overnight serum starvation, mature adipocytes (n = 4) were pre-treated with the indicate drug and stimulated with different insulin concentrations for 30 min at 37 °C, 5% CO_2_. 1.5 µCi of 2-Deoxy-D-^3^H-glucose (2DG) per ml were added at D-glucose 50 µM solution to incubate cells for 15 min, or indicated timing, at 37 °C. The test was terminated by ice treatment to block the glucose entry; adipocytes were lysed with NaOH 0.5 M and radioactivity measured by Scintillation counter (PerkinElmer, Waltham, MA, USA). An aliquot of each lysed well was collected to quantify protein concentration with Micro BCA Assay Kit for normalization.

### Cell viability assay

3 × 10^4^ 3T3-L1 cells were seeded in 96-well plates, differentiated using AdM as above described and then treated with CX-4945 at the reported conditions. 10^4^ human SVF cells were plated in 96-well plates and analyzed in the undifferentiated state (h-preadipocytes) while 2.5 × 10^4^ human SVF cells were assayed upon *in vitro* adipogenic differentiation (h-adipocytes) after CX-4945 treatment for 48 h at the indicated concentrations. MTT [3-(4,5-dimethylthiazol-2-yl)2,5-diphenyltetrazolium bromide] solution in PBS was added to each well (0.4 mg/ml) for 3 hours at 37 °C. Medium was removed and the formazan precipitate was dissolved in DMSO. OD was measured at 550 nm with a reference at 620 nm, using a Victor_3_^TM^ spectrometer (PerkinElmer); 6 replicates of each sample were assayed and each experiment was performed 3 times.

### Bodipy and Oil-Red-O staining and quantification

Cells were fixed for 1 h in 10% formalin/PBS at 4 °C and stained with Bodipy or Oil-Red O solution in 40% isopropanol, for 15 min at RT. After 3 washes with PBS, the fluorescence emission was immediately recorded at 513 nm, while the Oil-Red O staining was extracted in isopropanol, appropriately diluted and the absorbance was measured at 490 nm using Victor_3_^TM^.

### Cell and tissue lysis

Cells were lysed using a lysis buffer containing 20 mM Tris-HCl (pH 7.5), 1% Triton X-100, 10% glycerol, 1 mM EDTA, 150 mM NaCl and protease/phosphatase inhibitor cocktails for 1 h at 4 °C. Tissues were minced, covered by a lysis buffer containing 50 mM HEPES, pH 7.5, 150 mM NaCl, 10% glycerol, 5 mM Triton-X-100 and homogenized with a dounce homogenizer for 20 min. Cell lysates and tissue extracts were centrifuged (16000 g for 15 min) and protein concentration was determined in the supernatants by Bradford method.

### Western blot

Protein lysates were subjected to 11% or 15% SDS-PAGE, blotted on Immobilon-P membranes, incubated with the indicated antibodies and developed using an enhanced chemiluminescent detection system. Immunostained bands were quantified by means of a Kodak-Image-Station 4000MM-PRO and analyzed with Carestream Molecular Imaging software (New-Haven, CT, USA).

### CK2 kinase activity assay

Protein lysates were incubated for 10 min at 30^o^ C in 25 μl of a phosphorylation medium containing 50 mM Tris-HCl (pH 7.5), 100 mM NaCl, 12 mM MgCl_2_, 400 μM synthetic peptide-substrate RRRADDSDDDDD and 20 µM [γ-^33^P]ATP (1000 cpm/pmol). Assays were stopped by the absorption onto phosphocellulose filters. Filters were washed four times in 75 mM phosphoric acid^44^ and analyzed by a Scintillation Counter. Results were considered reliable when a linear correlation was observed between protein amounts added to the assays and CK2 activity.

### Subcellular fractionation by differential centrifugation

Plasma membrane (PM) and low density microsomes (LDM) were obtained using a differential centrifugation method previously described^46^. Briefly, 3T3-L1 mature adipocytes were washed and re-suspended in HES buffer (20 mM HEPES, pH 7.4, 1 mM EDTA, and 255 mM sucrose containing protease/phosphatase inhibitor cocktails). Cell lysates were prepared by shearing the cells through a 22-gauge needle 10 times and centrifugation at 19,000 g for 20 min at 4 °C. The pellet, containing the PM-rich fraction, was re-suspended in HES buffer and layered onto a 1.12 M sucrose cushion for centrifugation at 100,000 g for 60 min. The PM layer was removed from the sucrose cushion, centrifuged again at 40,000 g for 20 min and the resulting pellet was resuspended in lysis buffer. The supernatant obtained after the first centrifugation of lysates was further centrifuged at 41,000 g for 20 min and then at 100,000 g for 75 min to obtain the LDM pellet, which was re-suspended in lysis buffer.

### Immunolocalization of GLUT4 transporter by confocal microscopy

Cells were fixed in 4% paraformaldehyde, incubated with anti-GLUT4 antibody 1:50 for 2 h at RT, washed twice in PBS 1x and then incubated with a 488 donkey anti-goat IgG (H + L) (Thermo Fisher 1:200). Cells were counterstained with Draq5 (Abcam) and analyzed by a Leica TCS SP8 confocal microscope (Leica Microsystem, Hilden, Germany) using LAS X software (Leica). GLUT4 signal at the plasma membrane was quantified as described in Zhao *et al*.^47^.

### Mouse glucose and insulin tolerance tests

Insulin sensitivity was measured by GTT and ITT. Briefly, mice were fasted for 6 hours followed by ip injection of glucose (1.5 g/kg) or insulin (1U/kg) in saline solution. Blood was collected via tail tips at timed intervals (0, 15, 30, 60, 120 min) following injection. Glucose levels were measured using a glucometer (Freestyle Freedom Lite, Abbott, UK). Insulin levels were quantified with mouse Ultrasensitive Elisa Kit (ALPCO, Salem, NH, USA).

### RNA extraction and RT-real time PCR

Total RNA was extracted using RNeasy Mini Kits (QIAGEN, Hilden, Germany) following the supplier’s instructions. For each sample, 1 μg of RNA was treated with DNase Treatment & Removal Reagents (Thermo Fisher) and reverse-transcribed for 1 h at 37 °C with 150 ng random hexamers, 0.5 mM dNTPs, 20 units of RNAsin Ribonuclease Inhibitor and 200 units of M-MLV RT (Promega, Madison, WI, USA). Real Time PCR was carried out with SYBR Select MasterMix (Thermo Fisher) on an Applied Biosystems 7900HT Fast Real-Time PCR System. Duplicate samples (5 ng of cDNA) were normalized to the indicated reference gene and reported as arbitrary units ratio. Primer sequences and reaction conditions were reported in Supplementary Table S3.

### Statistics

Results are presented as mean ± SEM. Statistical significance was determined using unpaired Student’s t test (two-tailed). Differences were considered significant with p < 0.05.

### Study approval

Animal procedures were approved by the Animal Committee at Padua University (O.P.B.A.) (Permit Number: 56/2013), according to the “Guide for the Care and Use of Laboratory Animals”; all efforts were made to minimize animal suffering.

The Padua Ethical Committee for Clinical Research approved the study involving patients confirming that all methods were performed in accordance with the relevant guidelines and regulations (2892 P approved 10/06/2013); each subject gave informed written consent for AT biopsies.

### Data availability

The authors declare that the data supporting the findings of this study are available within the article and its supplementary information files.

## Electronic supplementary material

Supplementary Information
