# Supplementary material for: CK2 modulates adipocyte insulin-signaling and is up-regulated in human obesity
Source: Sci Rep. 2017 Dec 14;7:17569. doi: 10.1038/s41598-017-17809-w (PMC5730587; doi:10.1038/s41598-017-17809-w)
Supplement: Supplementary file 1 — Supplementary Information [file 41598_2017_17809_MOESM1_ESM.pdf]

Supplementary Information

CK2 modulates adipocyte insulin-signaling and is up-regulated in human obesity

Christian Borgo[1,6], Gabriella Milan[2,6,*], Francesca Favaretto[2], Fabio Stasi[2], Roberto Fabris[2,3], Valentina Salizzato[1], Luca Cesaro[1], Anna Belligoli[2,3], Marta Sanna[2,3], Mirto Foletto[3], Luca Prevedello[3], Vincenzo Vindigni[4], Romeo Bardini[5], Arianna Donella-Deana[1,*], Roberto Vettor[2,3]

[1]Department of Biomedical Sciences, University of Padua, 35131 Padua, Italy.

[2]Department of Medicine, University of Padua, Internal Medicine 3, 35128 Padua, Italy.

[3]Center for the Study and the Integrated Treatment of Obesity, Padua Hospital, 35128 Padua, Italy.

[4]Department of Neurosciences, University of Padua, 35128 Padua, Italy.

[5]Department of Surgical, Oncological and Gastroenterological Sciences, University of Padua, Division of General Surgery, 35128 Padua, Italy.

Additional footnotes

[6]Co-first author
[*]Corresponding authors

Corresponding authors

Prof. Arianna Donella-Deana
Department of Biomedical Sciences
University of Padua
via Ugo Bassi, 58b
35131 Padova, Italy
phone +39-049-8276110; fax +39-049-8276363
e-mail: arianna.donella@unipd.it

Dr. Gabriella Milan, BD, PhD
Department of Medicine
University of Padua
via Ospedale, 105
35128 Padova, Italy
phone +39-049-8218550; fax +39-049-8218555
e-mail: gabriella.milan@unipd.it

Supplementary Results

CK2 activity is not affected by insulin stimulation and can be pharmacologically inhibited in non-toxic conditions in murine and human adipocytes.

The potential effect of insulin stimulation on CK2 protein kinase was analyzed in 3T3-L1 mature adipocytes (Supplementary Fig. S1). After 1 h pre-incubation with DMSO (control) or two structurally unrelated CK2 inhibitors, CX-4945[28] and DMAT[29], cells were stimulated with 100 nM insulin and lysed. In controls (-) insulin treatment affected neither the protein-level of CK2α and CK2β subunits (Supplementary Fig. S1a) nor the activity of the protein kinase tested *in vitro* toward a CK2 specific peptide-substrate (Supplementary Fig. S1b). As expected, cell pre-treatment with CX-4945 or DMAT did not modify the cellular amount of CK2 subunits (Supplementary Fig. S1a), while it strongly reduced CK2 activity (Supplementary Fig. S1b).

Supplementary Fig. S2 shows that CX-4945 did not alter cellular viability of 3T3-L1 and human mature adipocytes treated in different culture conditions, with the exception of prolonged treatment (48 h) at the concentration of 10 μM in serum free medium. Moreover, Supplementary Fig. S3 demonstrates that the treatment of 3T3-L1 mature adipocytes with 2.5 μM CX-4945 for 3 h in serum free medium did not reduce the *Leptin*, *Adiponectin* and *Perilipin* mRNA level, while 10 μM CX-4945 for 24 h substantially inhibited the adipose-specific gene expression.

Additional experiments were performed differentiating murine and human pre-adipocytes in adipogenic medium containing non-cytotoxic low CX-4945 concentration as indicated in Supplementary Fig. S2. Under these conditions, CK2 activity was strongly inhibited as demonstrated in cells by the abrogated phosphorylation of the CK2-target Akt1 Ser129 (Supplementary Figs. S4b and S5b) and *in vitro* toward a CK2-specific peptide-substrate (Supplementary Figs. S4c and S5c). Our experiments showed that CK2-inhibition did not

interfere with morphology, intracellular lipid content and adipogenic gene expression profile of murine (Supplementary Fig. S4a,d,e) or human adipocytes (Supplementary Fig. S5a,d,e), differently from what described by other authors using higher concentrations of CK2-inhibitors[48,49,50].

Supplementary References

48. Wilhelm N, Kostelnik K, Götz C, Montenarh M. Protein kinase CK2 is implicated in early steps of the differentiation of pre-adipocytes into adipocytes. *Mol. Cell. Biochem.* **365**(1–2):37–45 (2012).

49. Schwind L, Wilhelm N, Kartarius S, Montenarh M, Gorjup E, Gotz C. Protein kinase CK2 is necessary for the adipogenic differentiation of human mesenchymal stem cells. *Biochim. Biophys. Acta* c**1853**(10 Pt A):2207–2216 (2015).

50. Schwind L, Nalbach L, Zimmer AD, Kostelnik KB, Menegatti J, Grässer F, Götz C,Montenarh M. Quinalizarin inhibits adipogenesis through down-regulation of transcription factors and microRNA modulation. *Biochim Biophys Acta*. Dec;**1861(**12):3272-3281 (2017).

Supplementary Figures and Figure Legends

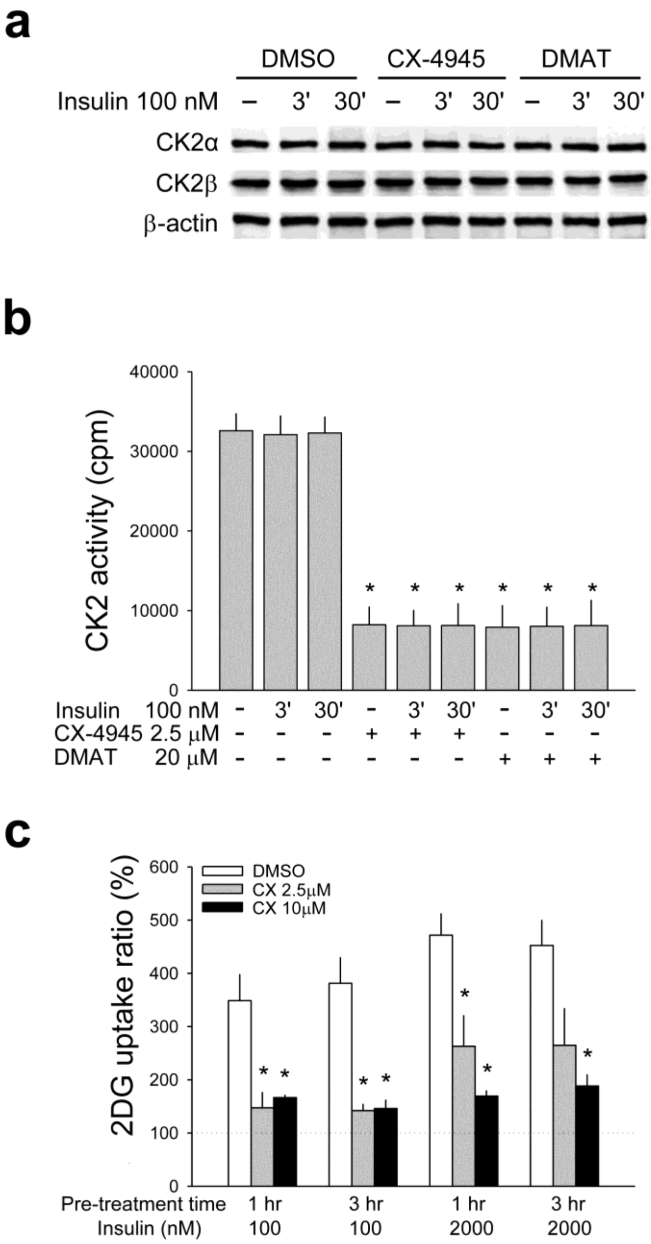

Supplementary Fig. S1. Insulin treatment of 3T3-L1 adipocytes does not affect CK2 expression and activity whereas CK2 inhibitors reduce the kinase activity and glucose uptake in a dose/time dependent manner. (**a**,**b**) After overnight serum starvation 3T3-L1 adipocytes (n=3) were pre-incubated with vehicle (DMSO), 2.5 μM CX-4945 or 20 μM DMAT for 1 h and then stimulated or not (-) with 100 nM insulin for 3 or 30 min. (**a**) Lysate proteins (40 μg) were analyzed by Wb with anti-CK2α and anti-CK2β antibodies. β-actin is shown as loading control. Panel is representative of four separate experiments. (**b**) Lysate proteins (2 μg) were tested for CK2 kinase activity. Reported values are means ± SEM of four separate experiments. *$p<0.05$ *vs* untreated cells. (**c**) Insulin-induced 2DG-uptake of 3T3-L1 adipocytes (n=4), pre-treated with DMSO or CX-4945 for the indicated times and stimulated for 30 min with insulin. Data are normalized for total protein content and reported as glucose uptake ratio in percentage (%) over basal uptake (untreated with insulin), which was set as 100 and indicated by dotted line. Panel is representative of at least 4 separate experiments. *$p<0.05$ *vs* DMSO. Results are presented as mean ± SEM.

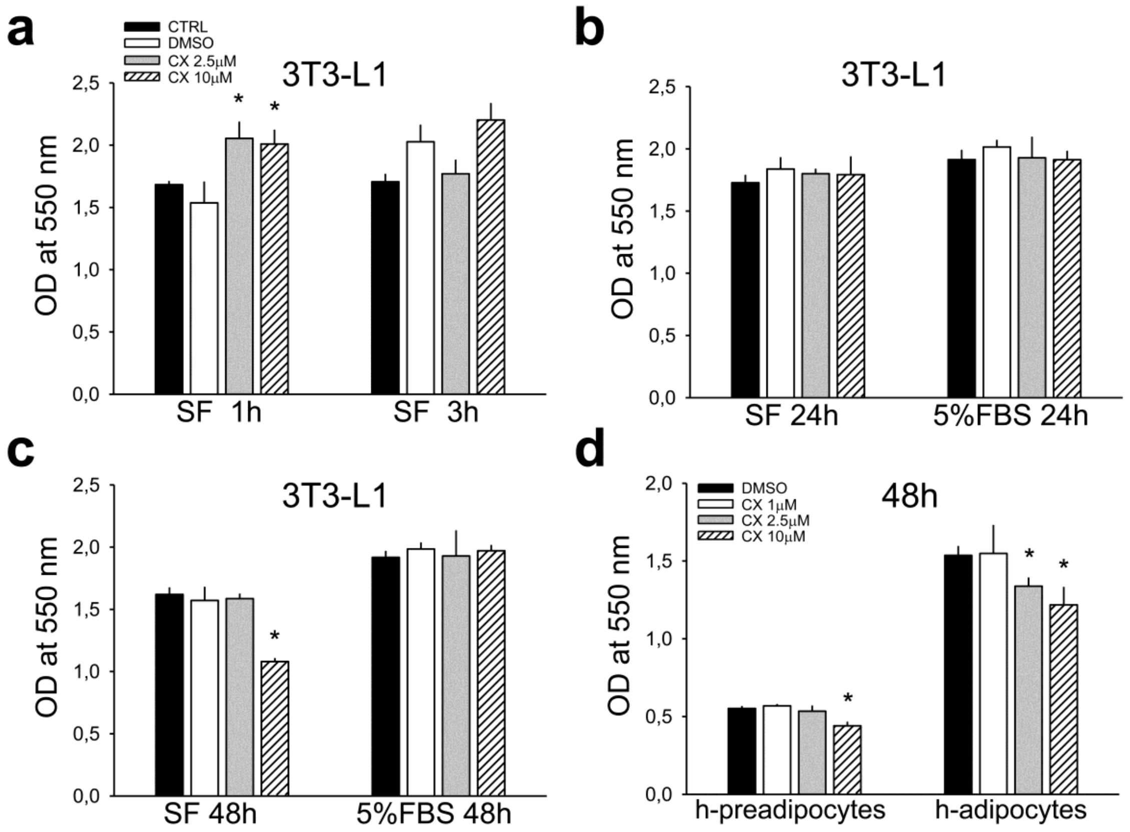

Supplementary Fig. S2. Cell viability assay of 3T3-L1 adipocytes, human pre-adipocytes and adipocytes treated with CX-4945 in different culture conditions. (**a**,**b**,**c**) *In vitro* differentiated 3T3-L1 adipocytes (n=6) were untreated (CTRL), treated with DMSO or CX-4945 in serum free culture medium (SF) for 1 or 3 h (**a**) (to reproduce the experimental conditions used in the experiments presented in Fig. 1, 2 and S1), for 24 h (**b**) and for 48 h (**c**) with or without 5% FBS. (**d**) Human (h) pre-adipocytes (n=6), obtained from SAT, and *in vitro* differentiated mature adipocytes were treated for 48 hours with DMSO or CX-4945 in medium with 10% FBS and in SF, respectively. (**a-d**) Cell viability was assessed by MTT colorimetric test and absorbance values are reported. *$p<0.05$ *vs* CTRL or DMSO. Similar results were obtained in at least 3 independent experiments. Results are presented as mean ± SEM.

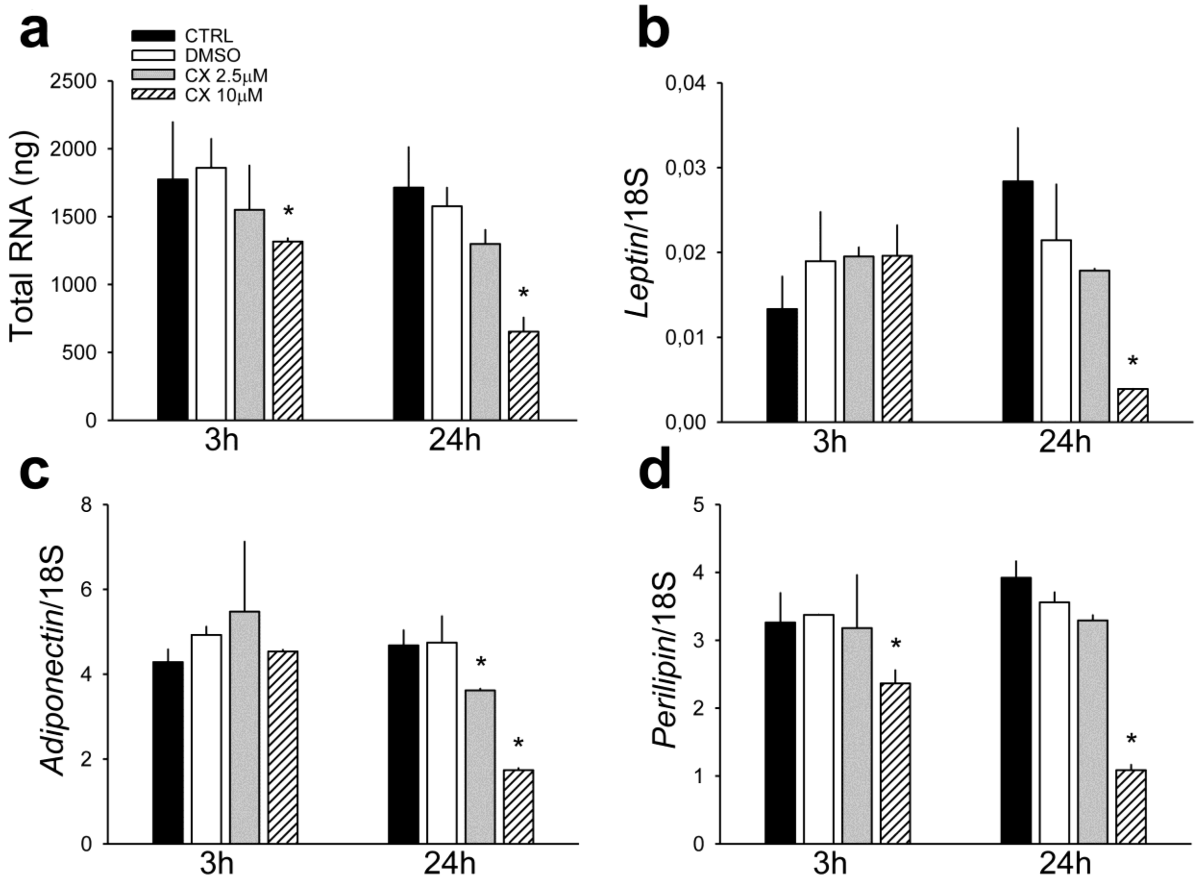

Supplementary Fig. S3. *Leptin, Adiponectin* and *Perilipin* mRNA expression upon 3/24 hour-CX-4945 treatment in 3T3-L1 adipocytes. (**a-d**) Total RNA (**a**) and mRNA expression of *Leptin* (**b**), *Adiponectin* (**c**) and *Perilipin* (**d**) were quantified in 3T3-L1 adipocytes differentiated *in vitro* (n=2) untreated (CTRL) or treated with DMSO or CX-4945 in serum free culture medium for 3 or 24 hours. Expression data were normalized by 18S rRNA (18S) content and reported as arbitrary unit ratio. Similar results were obtained in at least 2 independent assays. *p<0.05 *vs* CTRL. Results are presented as mean ± SEM.

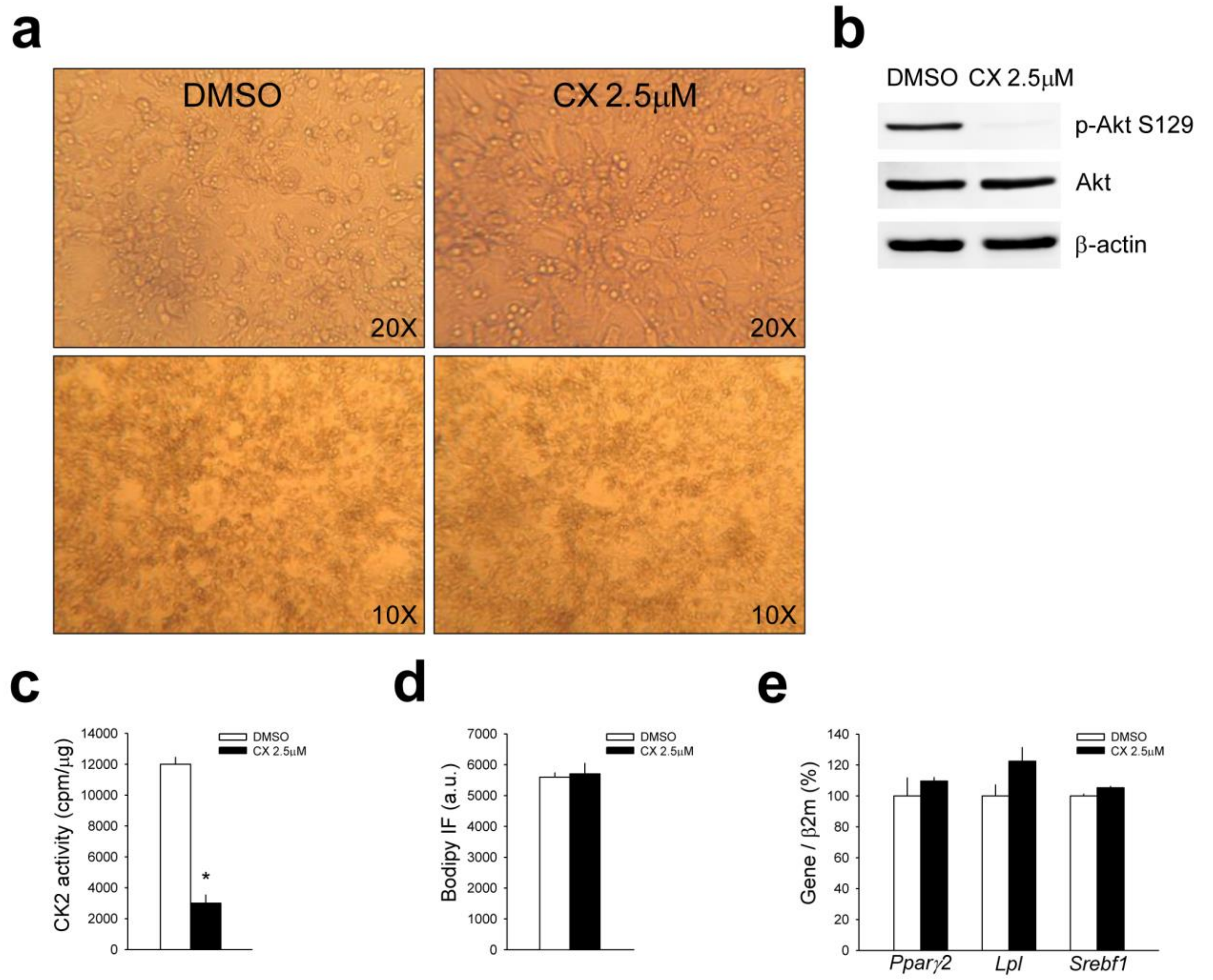

Supplementary Fig. S4. CX-4945 treatment does not influence *in vitro* adipogenic differentiation of murine pre-adipocytes. (**a-d**) 3T3-L1 pre-adipocytes were pre-treated for 1 h and differentiated for 8 days in Adipogenic Medium (AdM) containing DMSO or CX-4945, changing the medium every 3 days. (**a**) The panel, representative of 3 independent experiments, shows images of the resulting mature adipocytes (20X and 10X magnification). (**b**) 3T3-L1 mature adipocytes were homogenized and protein lysates (40 μg) were analyzed by Wb with anti-p-Akt S129 and anti-Akt antibodies; anti-β-actin Wb is shown as loading control. Panel is representative of 3 independent experiments. (**c**) CK2 activity was quantified in cell lysates (n=4) obtained from 3T3-L1 adipocytes and expressed as ^{33}P-phosphate transferred to the substrate per μg of protein lysates. *p<0.05 *vs* DMSO. (**d**) Intracellular lipids of 3T3-L1 adipocytes (n=4) were stained by 488-Bodipy and the intensity of fluorescence (IF) was quantified by Victor 3. Results are presented as mean ± SEM. (**e**) The expression level of *Pparγ2*, *Lpl* and *Srebf1* mRNA was quantified by RT-qPCR in 3T3-L1 pre-adipocytes (n=3) treated for 3 days in AdM with DMSO or CX-4945, normalized to *β2 microglobulin* mRNA (β2m) level and reported as percentage (%) relative to control (DMSO).

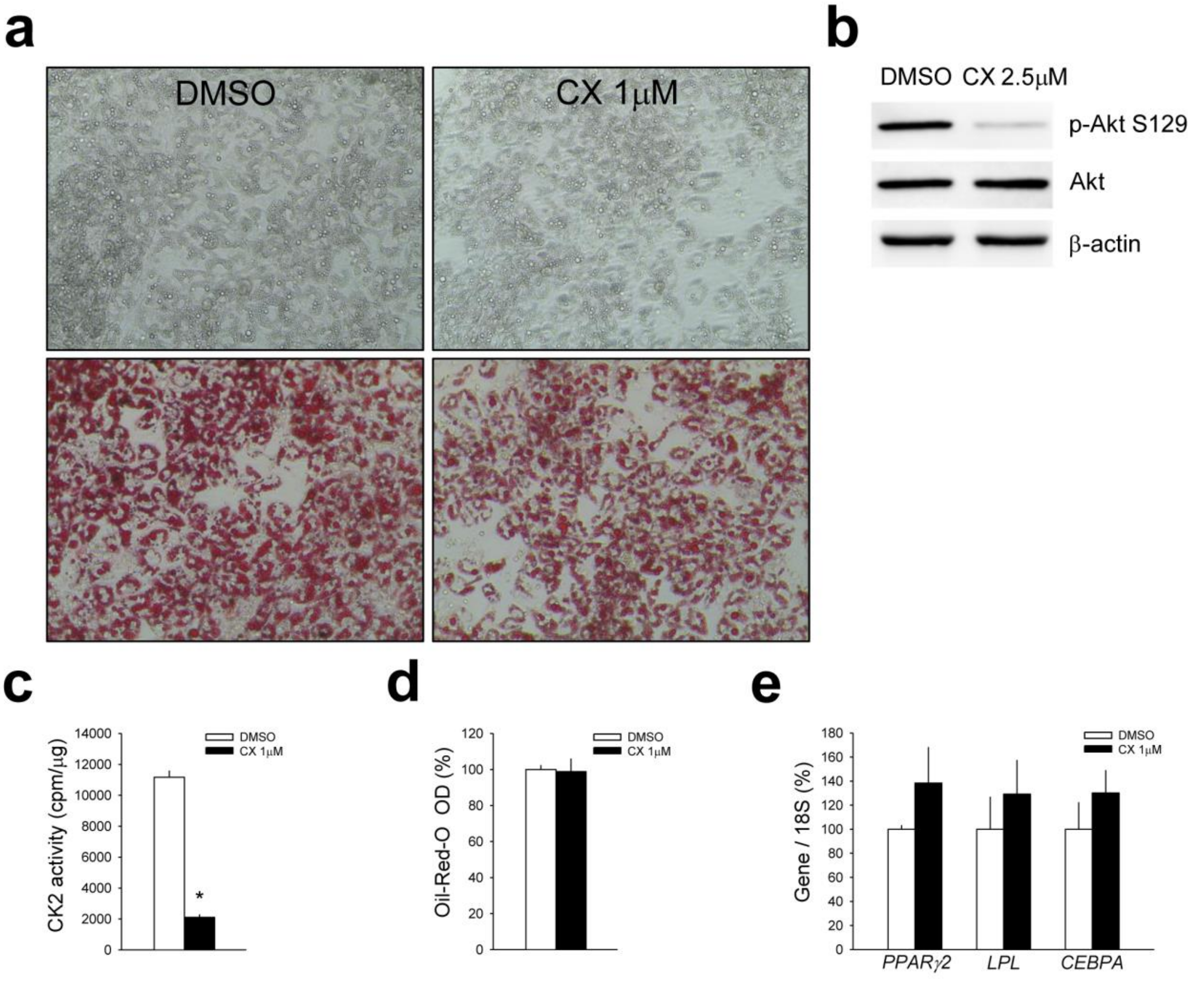

Supplementary Fig. S5. CX-4945 treatment does not influence *in vitro* adipogenic differentiation of human primary pre-adipocytes. (**a-e**) Human primary cultures of pre-adipocytes, obtained from SAT of two patients (5806 and 5860), were differentiated for 13 days in h-AdM with DMSO or 1 μM CX-4945 (CX), changing the medium every 3 days. (**a**) Representative images of mature adipocytes at phase contrast (upper panels) and upon Oil-Red-O staining (lower panels) (20X magnification). (**b**) Human adipocytes were homogenized and protein lysates (40 μg) were analyzed by Wb with anti-p-Akt S129 and anti-Akt antibodies; β-actin is shown as loading control. Panel is representative of three independent experiments. (**c**) CK2 activity was quantified in cell lysates (n=4) obtained from human adipocytes and expressed as ^{33}P-phosphate transferred to the substrate per μg of protein lysates. *$p<0.05$ *vs* DMSO. (**d**) Intracellular lipids of human primary adipocytes (n=4) were stained by Oil-Red-O and the optical density (OD) was quantify at 490 nm. Results are presented as mean ± SEM. (**e**) The expression level of *PPARγ2*, *LPL* and *CEBPA* mRNA was quantified by RT-qPCR in human adipocytes (n=4), normalized to 18S rRNA (18S) content and reported as percentage (%) relative to control (DMSO).

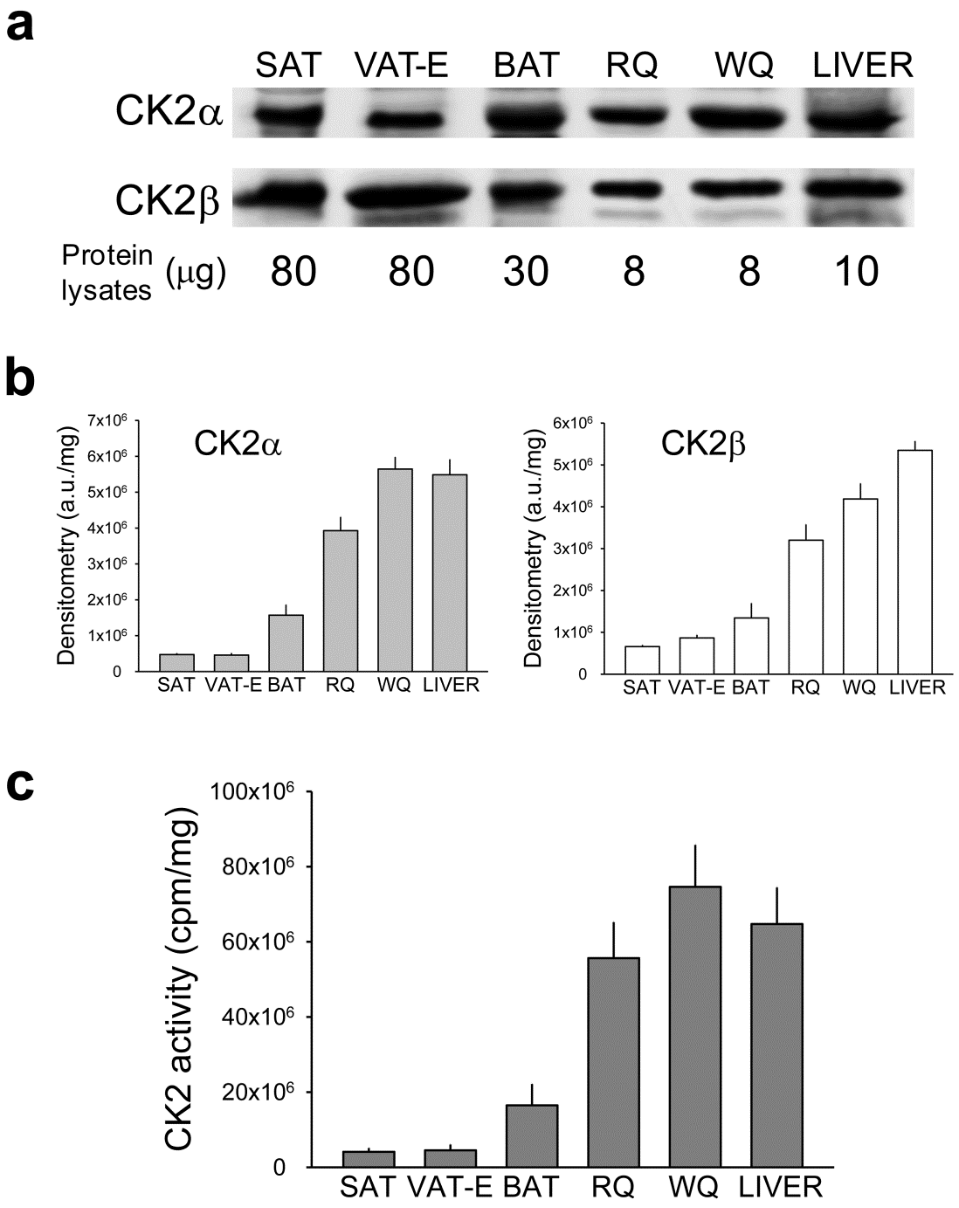

Supplementary Fig. S6. CK2 expression and activity in insulin target tissues of B6 male mice. (**a**, **b** and **c**) C57BL6/6J (B6) male mice (n=3) of 22 weeks of age were starved for 6 h and sacrificed to collect the following tissues: subcutaneous (SAT), visceral epididimal (VAT-E) and brown (BAT) adipose tissues, red (RQ) and white quadriceps (WQ), and liver. (**a**) The indicated amounts (μg) of tissue proteins were analyzed by Wb with anti-CK2α and anti-CK2β antibodies. (**b**) Immunostained bands were quantified by densitometry and expressed as arbitrary units/mg proteins. Results are presented as mean ± SEM. **CK2α**: SAT *vs* BAT p=0.0047; VAT *vs* BAT p=0.0041; BAT *vs* RQ p=0.000093; RQ *vs* WQ p=0.00054; RQ *vs* LIVER p=0.0016; LIVER *vs* BAT p=0.000014. **CK2β**: SAT *vs* VAT p=0.006; VAT *vs* BAT p=0.07; SAT *vs* BAT p=0.03; BAT *vs* RQ p=0.00036; RQ *vs* WQ p=0.0098; RQ *vs* LIVER p=0.00023 ; WQ *vs* LIVER p=0.0032; LIVER *vs* BAT p=0.0000064. (**c**) CK2 activity was quantified in tissue lysate proteins of mice (n=3) and expressed as ^{33}P-phosphate transferred to the substrate per mg of lysate proteins. Statistical analysis: SAT *vs* BAT p=0.021; VAT *vs* BAT p=0.021; BAT *vs* RQ p=0.00098; RQ *vs* WQ p=0.042; RQ *vs* LIVER p=0.23; WQ *vs* LIVER p=0.23; LIVER *vs* BAT p=0.00042; WQ *vs* BAT p=0.0043. Panels are representative of at least 4 separate experiments.

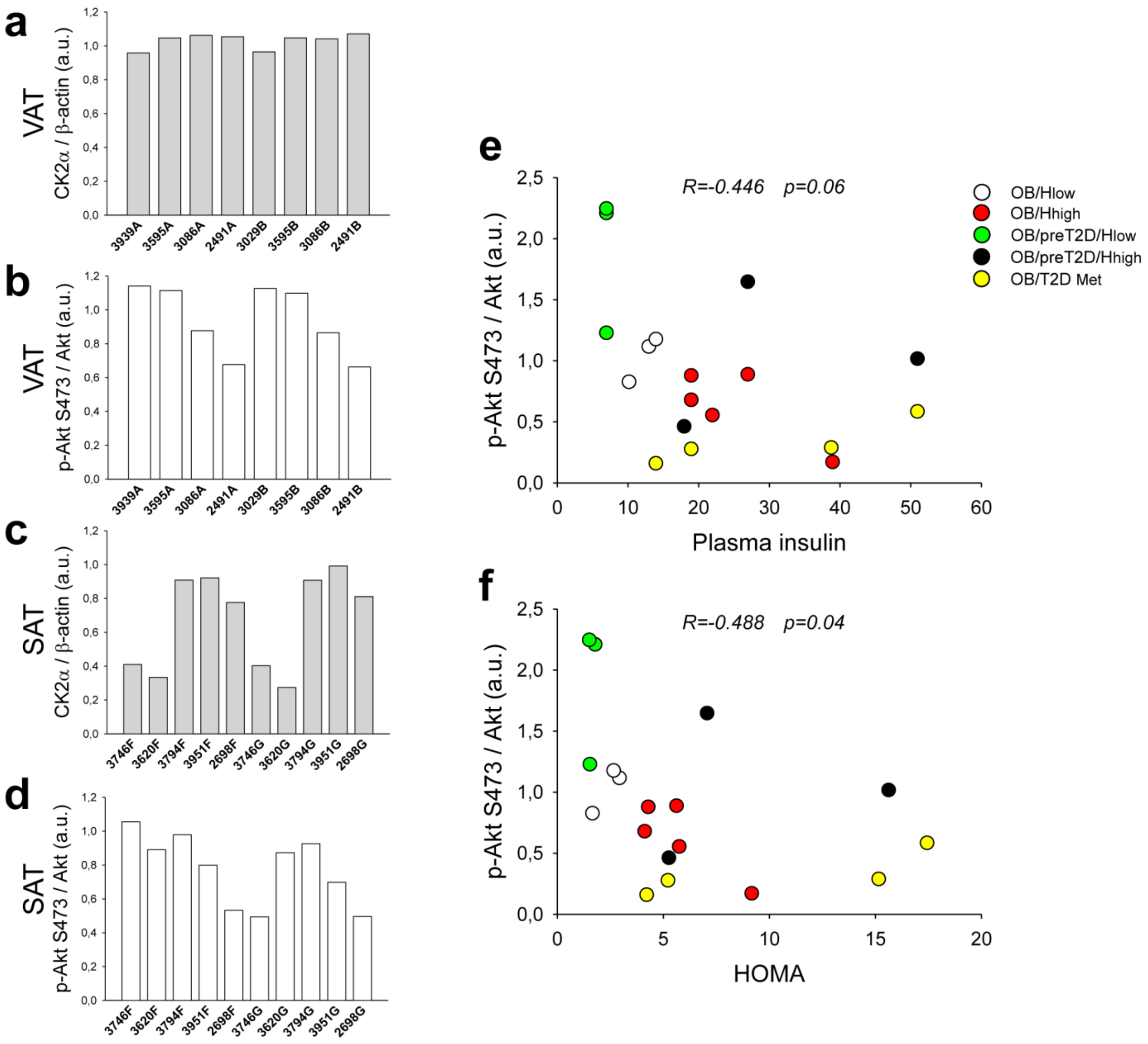

Supplementary Fig. S7. Reference samples and correlation between VAT Akt phosphorylation and clinical parameters. (**a-d**) Comparison between the values of VAT (**a**, **b**) and SAT (**c**, **d**) reference samples analyzed in Fig. 7 (panels a, b and f, g, respectively). Panels demonstrate that the reference samples loaded in two separate gels (Fig. 7) show similar values relative to the CK2α amount (**a**, **c**) and Akt S473 phosphorylation extent (**b**, **d**) that were quantified by densitometry and expressed in arbitrary units (a.u.). Samples are specified by the patient ID number followed by a letter indicating the Fig. 7 panel, in which they are shown. (**e**, **f**) Plots represent the correlation between VAT Akt phosphorylation extent, estimated by the densitometric analysis of Wb shown in Fig. 7a,b and the fasting insulin level (**e**) or the HOMA index (**f**) of obese patients divided in different subgroups according to the clinical evaluations reported in Supplementary Table S1. Statistical analysis was performed by Spearman's rank correlation coefficient calculation.

Supplementary Tables

Group	ID	Sex	Age	Glycemic profile	DM2 therapy	Weight	BMI	Waist	Glucose	Insulin	HOMA	IL-6	TNFα	hsCRP	Leptin
**OB**	2432	F	47	N		111.0	42.82	114	5.0	12.0	2.67	1.9	9.1	2.08	64.0
	2740	F	41	preT2D		156.0	53.98	149	6.2	20.0	5.51	1.9	6.8	7.61	55.7
	2476	F	41	N		118.0	46.68	119	5.2	2.0	0.46	7.1	8.5	8.47	43.0
	2980	M	46	N		143.0	50.07	135	4.8	5.0	1.07	1.9	5.5	7.12	20.0
**OB/H low**	2352	M	67	N		123.0	43.07	131	3.7	10.2	1.68	2.5	6.9	0.49	17.0
	3939	M	50	N		126.0	38.46	123	4.7	3.0	0.63	5.8	8.1	2.30	nd
	3595	F	40	N		106.0	38.01	118	5.1	13.0	2.95	2.0	8.4	0.98	29.0
	3970	F	22	N		128.0	48.18	135	4.3	14.0	2.68	2.1	5.8	>10	50.0
**OB/H high**	3086	F	37	N		128.0	45.90	110	5.1	19.0	4.31	1.9	7.0	4.19	36.0
	2491	M	37	N		148.7	46.41	125	4.9	19.0	4.14	1.9	9.0	1.32	14.0
	2492	F	36	N		120.0	44.62	117	4.7	27.0	5.64	1.9	7.1	7.59	35.0
	3726	F	41	N		117.0	45.14	126	5.6	22.0	5.48	3.1	9.8	>10	36.0
	3502*	F	33	N		120.0	44.08	142	5.3	39.0	9.19	4.5	8.2	19.20	58.0
**OB/preT2D/H low**	3794	F	59	preT2D		100.0	41.62	124	5.8	7.0	1.80	2.0	nd	3.81	41.0
	4044	F	65	preT2D		124.0	59.80	135	5.0	7.0	1.56	5.8	9.2	>10	50.0
	3921	F	31	preT2D		120.0	38.74	125	4.9	7.0	1.52	2.0	8.9	>10	16.0
**OB/preT2D/H high**	3951	M	37	preT2D		116.0	39.21	128	5.9	27.0	7.08	2.9	4.0	2.29	16.0
	3843	M	63	preT2D		121.0	40.43	133	6.9	51.0	15.64	2.0	8.2	4.01	33.0
	3585	F	57	preT2D		138.0	51.31	143	6.6	18.0	5.28	3.0	8.9	19.5	42.0
**OB/T2D**	2862	F	64	T2D	Ins	130.0	45.52	142	8.6	nd	nd	1.9	10.5	3.15	43.0
	2597	F	54	T2D	Met+SU	92.0	38.29	123	5.1	2.0	0.45	10.0	12.0	2.18	32.0
	2267	M	58	T2D	Met+Ins	147.0	53,35	145	12.0	nd	nd	2.8	7.5	10.10	19.0
	3256	M	54	T2D	Ins	160.0	49.94	157	nd	nd	nd	6.1	10.8	30.20	59.0
**OB/T2D Met**	2698	F	56	T2D	Met	115.0	43.28	132	6.8	14.0	4.23	2.4	7.1	19.60	39.0
	2540	M	56	T2D	Met	122.0	39.84	140	8.8	38.8	15.18	1.9	8.6	8.41	26.0
	2650	F	49	T2D	Met	136.0	48.19	136	6.2	19.0	5.24	2.4	6.6	13.20	43.0
	3110	M	62	T2D	Met	114.0	38.53	142	7.7	51.0	17.45	2.0	13.1	4.83	43.0

*patient affected by HAIR-AN syndrome characterized by hyperandrogenism, insulin resistance and acanthosis nigricans

Supplementary Table S1. Anthropometric, metabolic and inflammatory parameters in the 27 studied obese patients. Each patient was identified by an identification number (ID). Sex (F=female, M=male) and age (years) are indicated. Patients were classified as normoglycemic (N), prediabetic (preT2D) or type 2 diabetic (T2D) on the basis of America Diabetes Association, Standards of Medical Care in diabetes-2016 (Diabetes Care 2016;39(Suppl. 1):S1–S2|DOI: 10.2337/dc16-S001) (Glycemic profile). The anti-diabetic therapy of diabetic patients is reported indicating insulin or non-insulin hypoglycemic drug treatment (Ins=insulin; SU=sulfonylureas; Met=metformin). Weight (kg), body mass index (BMI, Kg/m^2) and waist (cm) are obtained by clinical anthropometric evaluation. Fasting blood glucose (mmol/l), insulin (mU/l) and HOMA (=fasting glucose x fasting insulin/22.5) are reported as metabolic characterization. Inflammatory profile was assessed measuring blood levels of IL-6 (ng/l), TNF-α (ng/L) and hsCRP (=high-sensitive C-reactive Protein, mg/L); blood level of the adipokine Leptin (μg/l) was quantified.

OB=obese
OB/H low=obese normoglycemic with HOMA <3
OB/H high=obese normoglycemic with HOMA>3
OB/preT2D/H low=obese prediabetic with HOMA<3
OB/preT2D/H high=obese prediabetic with HOMA>3
OB/T2D=obese diabetic
OB/T2D Met=obese diabetic in therapy with metformin (oral anti-diabetic drug)

Obese normoglycemic (OB/N, n=12) obese prediabetic (OB/preT2D, n=7) and obese diabetic (OB/T2D; n=8). No statistical differences were observed in the 3 groups between mean values of weight (124.1±12.1; 125.0±17.7; 127.0±21.2), BMI (44.45±3.16; 46.44±8.46; 44.62±5.60) and Leptin level (36.6±16.3; 36.2±15.6; 38.0±12.3). OB/T2D group displayed a mean value of age (57±5 *vs* 41±11, p=0.0006) and waist (139.6±9.9 *vs* 124.5±9.6, p=0.004) higher than OB group; OB/preT2D group had an intermediate mean age (51±14) and waist (133.9±9.3) not significantly different from the other groups.
The 3 groups differed significantly in fasting blood glucose level (4.9±0.6; 5.9±0.7; 7.9±2.2; $p<0.05$) as expected by the selection criteria used.
In regard to systemic inflammation markers no statistical differences were obtained, but in OB, OB/preT2D and OB/T2D groups an increase trend was observed for IL-6 (3.0±1.8; 2.8±1.4; 3.7±2.9), TNF-α (7.8±1.3; 7.7±2.0; 9.5±2.4) and hsCRP (5.37±5.68; 7.44±7.02; 11.5±9.5) levels.
OB and the OB/preT2D groups were further divided in 2 subgroups on the basis of the insulin resistance degree estimated by HOMA index (HOMA low = HOMA<3 and HOMA high = HOMA>3).
Considering all patients with low HOMA (n=10) *vs* patients with high HOMA or diabetes (T2D) (n=17), independently of their glucose profile, they did not significantly differ in age, weight, BMI and Leptin level, but showed a statistical different mean value of waist (125.8±7.7 *vs* 134.7±12.1, p=0.03), fasting blood glucose (4.9±0.6 *vs* 6.7±1.9 p=0.002), hsCRP level (3.61±3.07 *vs* 9.84±8.32 p=0.02) and, as expected for the selection criteria used, blood fasting insulin (8.0±4.1 *vs* 26.2±14.0, p=0.0003) and HOMA index (1.70±0.86 *vs* 7.51±5.03 p=0.0008).

Group	ID	Sex	Age	Glycemic profile	Surgery	Weight loss intervention	Weight PRE	Weight POST	Delta Weight	BMI pre	BMI post	Delta BMI	% EWL
**OB/WL**	3406	F	40	N	Cholecystectomy	SG	106.0	77.0	-29.0	38.93	28.28	-10.65	76.4
	2370	F	39	N	Cholecystectomy	SG	124.7	75.0	-49.7	51.90	31.22	-20.69	76.9
	2377	F	58	N	Cholecystectomy	SG	175.0	99.0	-76.0	63.51	35.93	-27.58	71.6
**OB/WL ps**	2664	F	33	nd	Abdominoplasty	GB	176.0	113.0	-63.0	57.47	36.90	-20.57	63.4
	2441	F	67	T2D	Abdominoplasty	diet	120.0	98.0	-22.0	45.17	36.89	-8.28	41.1
	748	M	56	N	Abdominoplasty	GB	125.0	53.0	-72.0	41.29	23.78	-17.51	107.5
	3519	F	34	N	Abdominoplasty	GB	114.0	84.0	-30.0	40.39	29.76	-10.63	69.1
	2785	F	47	ex-T2D	Abdominoplasty	SG	101.0	68.5	-32.5	38.96	26.43	-12.54	89.8
	2087	F	58	N	Abdominoplasty	SG	102.0	70.0	-32.0	38.87	26.67	-12.19	87.9
	4098	F	38	N	Abdominoplasty	diet	90.0	58.0	-32.0	36.51	23.53	-12.98	112.8
	4208	F	27	N	Abdominoplasty	diet	125.0	76.0	-49.0	40.82	24.82	-16.00	101.2
	3132	F	50	N	Abdominoplasty	SG	101.0	60.6	-40.4	37.55	22.53	-15.02	119.7

Supplementary Table S2. Clinical characteristics of obese patients after weight loss. Each patient was identified by an identification number (ID). Sex (F=female, M=male) and age (years) are indicated. Patients were classified as normoglycemic (N), prediabetic (preT2D) or diabetic (T2D) as reported in Supplementary Table S1 (Glycemic Profile). Ex-T2D indicates diabetes regression after weight loss. SAT and VAT biopsies were collected during cholecystectomy, while only SAT biopsies during plastic surgery (abdominoplasty). Weight loss (WL) was obtained by bariatric surgery (SG=sleeve gastrectomy, GB= gastric banding) or caloric restriction (diet). Weight (kg) and body mass index (BMI, Kg/m^2) before (PRE) and after (POST) WL-intervention were reported as well as their differences (delta). The percent of excess weight loss (% EWL= [(preoperative weight-current weight)/ preoperative weight-ideal weight]x100) was used to estimate the degree of WL and the efficacy of WL-intervention.
After WL-intervention all patients reduced significantly their weight (121.6±27.6 *vs* 79.3±16.5 $p<0.001$) and their BMI (44.28±8.69 *vs* 28.89±5.28 $p<0.0001$) with a mean value of 84.8% EWL.

GENE	FORWARD (5'-3')	REVERSE (5'-3')	ANNEALING (°C)	PRIMER (F/R nM)	AMPLICON (bp)
PPARγ2	ACCCAGAAAGCGATTCCTTCA	AGTGGTCTTCCATTACGGAGAGATC	60	900/900	87
LPL	GCACCTGCGGTATTTGTGAA	TGAAACACCCCAAACACTGG	60	300/300	160
CEBPA	TGGAAATGCAAACTCACCGC	TGCTGTAGCCTCGGGAAGG	60	300/300	104
Pparγ2	TTCGCTGATGCACTGCCTATGA	GAATGCGAGTGGTCTTCCATCA	60	300/300	128
Lpl	TCAGAGCCAAGAGAAGCAGCAA	TGTGTTGCTTGCCATCCTCA	60	300/300	117
Srebf1	ACGGAGACAGGGAGTTCTCA	TGCTGGAGCTGACAGAGAAA	60	300/300	105
Leptin	TCCAGAAAGTCCAGGATGACAC	CACATTTTGGGAAGGCAGG	60	300/300	211
Adiponectin	ACAATGGCACACCAGGCCGTGA	AGCGGCTTCTCCAGGCTCTCCTTT	58	300/300	179
Perilipin	GAAGCATCGAGAAGGTGGTAGAGTTC	TGCTGTGGTTTGCATGGTGT	60	300/300	167
**β2m**	ACCCTGGTCTTTCTGGTGC	TTTTTTTCCCGTTCTTCAGC	56	300/300	181
**18S**	CGGCTACCACATCCAAGGAA	GCTGGAATTACCGCGGCT	60	100/100	186

Supplementary Table S3. Primer sequences and qPCR conditions. Primer sequences, annealing temperature, working concentration and amplicon size were reported for each mRNA quantified by real-time PCR. Human sequences: *PPARγ2*= peroxisome proliferator-activated receptor gamma, isoform 2; *LPL*= lipoprotein lipase; *CEBPA*= CCAAT/enhancer binding protein α. Mouse sequences: *Pparγ2*; *Lpl*; *Srebf1*=sterol regulatory element binding transcription factor 1; *Leptin*; *Adiponectin*, *Perilipin*. As reference we used β2m (*β2 microglobulin*) for mouse targets and 18S (18S ribosomal RNA) for both human and mouse targets.
